# Reinforced PHA/CNC Biocomposites in Extrusion-Based Additive Manufacturing

**DOI:** 10.1021/acsomega.5c05743

**Published:** 2025-08-05

**Authors:** Markos Petousis, Constantine David, Dimitrios Sagris, Nektarios K. Nasikas, Vassilis Papadakis, Apostolos Argyros, Vasileios Stratiotou Efstratiadis, Aikaterini Gaganatsiou, Nikolaos Michailidis, Nectarios Vidakis

**Affiliations:** † Department of Mechanical Engineering, 112178 Hellenic Mediterranean University, Heraklion 71410, Greece; ‡ Department of Mechanical Engineering, 125444 International Hellenic University, Serres Campus, Serres 62124, Greece; § Division of Mathematics and Engineering Sciences, Department of Military Sciences, 69139 Hellenic Army Academy, 16673 Vari, Attica, Greece; ∥ Institute of Electronic Structure and Laser of the Foundation for Research and Technology-Hellas (IESL-FORTH) − Hellas, N. Plastira 100m, 70013 Heraklion, Greece; ⊥ Department of Industrial Design and Production Engineering, University of West Attica, 122 43 Athens, Greece; # Physical Metallurgy Laboratory, Mechanical Engineering Department, School of Engineering, 37782 Aristotle University of Thessaloniki, 54124 Thessaloniki, Greece; ∇ Centre for Research & Development of Advanced Materials (CERDAM), Centre for Interdisciplinary Research and Innovation, Balkan Centre, Building B’, 10th km Thessaloniki-Thermi Road, 57001 Thessaloniki, Greece

## Abstract

Polyhydroxyalkanoate (PHA) is a biopolymer that can be 3D printed using the material extrusion method. Nevertheless, their mechanical properties are inferior to those of petroleum-derived polymers, which restricts their broader application. Herein, nanobiocomposites comprising naturally sourced PHA and cellulose nanocrystals (CNC) as fillers were successfully synthesized. These nanobiocomposites were prepared with filler concentrations ranging from 0.5 to 2.5% by weight, in increments of 0.5 wt %. Filaments were produced from binary PHA/CNC mixtures and subsequently employed for 3D printing of the respective nanobiocomposite samples. They were subjected to mechanical, rheological, thermal, and structural analyses using analytical techniques and the respective standards. The integration of CNC into the pure PHA polymer matrix has been reported to enhance PHA’s mechanical properties of PHA, with an increase in flexural strength by 23.3%, flexural modulus by 20.8%, and Young’s modulus by 47.3%, although there was a reduction in impact strength and microhardness. Morphological characterization confirmed the homogeneous dispersion of CNC, whereas the thermal and rheological properties remained almost unchanged. The porosity and geometric accuracy of the 3D-printed samples, evaluated using micro-CT, were improved by incorporating CNC into the PHA matrix. The 0.5 wt % CNC concentration was the optimum one, improving mechanical and quality metrics. These findings highlight the potential of PHA/CNC nanocomposites as innovative high-performance biodegradable materials for 3D printing for biomedical, packaging, and structural engineering applications. Such nanobiocomposites can contribute to reducing the environmental impact of petroleum polymers through a cost-effective additive manufacturing method.

## Graphical abstract

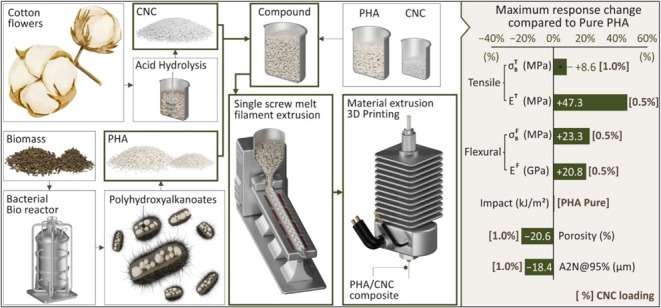

## 1 Introduction

In a rapidly evolving technological world, the race for novel, multifunctional, and robust materials has recently accelerated through the development of Additive Manufacturing (AM) processes.^1–7^ The utilization of AM materials and their respective multifunctionalities ranges from biocompatibility^8–10^ for relevant medically related applications to enhanced mechanical properties^11,12^ for applications that require enhanced performance and sustainability. The most utilized materials in the above context are polymers because of their intriguing properties such as relatively low melting points,^13^ easy manufacturing processes,^14^ and recyclability.^15^ Other materials that are commonly used in AM include metals,^16^ ceramics,^17^ and glass.^18^

AM, generally termed 3D printing, covers a broad spectrum of relevant techniques and^19^ thus plays a crucial role in the development of new possibilities for materials and technologies. These possibilities pave the way for addressing acute problems, such as climate change,^19^ economic crises,^20^ and pandemics.^21^ Recent studies of multifunctional materials prepared using AM have focused on biocidal activity through the incorporation of antibacterial nanoparticles into various polymeric matrices. These include polypropylene/Ag nanoparticle composites^22^ and biomedical-grade isotactic polypropylene.^23^ Concomitantly, the combination of antibacterial properties with the enhancement of their mechanical properties is also of great importance, as they are frequently used as biomaterials exhibiting antimicrobial properties combined with the enhancement of their mechanical properties.^22–25^

One of the most utilized AM techniques is Material Extrusion (MEX),^26^ in which the material, in the form of a filament, is fed into an extruder and subsequently deposited as a melt in successive layers, resulting in a three-dimensional object upon cooling. Vat photopolymerization (VPP) is another common AM technique in which a specific light source is selected to selectively solidify a liquid resin, which in turn produces a three-dimensional object.^27,28^

As previously mentioned, the most commonly used materials in AM are polymers with a variety of chemical and physical characteristics. These polymers include polylactic acid (PLA),^29^ polycarbonate (PC),^30,31^ acrylonitrile butadiene styrene (ABS),^32^ polyethylene terephthalate glycol (PETG),^33^ polyethylene terephthalate (PET),^34^ thermoplastic polyurethane (TPU),^35^ polypropylene (PP),^36,37^ polyamide,^38^ high-performance poly­(ether imide) (ULTEM),^39^ acrylonitrile styrene acrylate (ASA),^15^ poly­(methyl methacrylate)^12^ and high-density polyethylene (HDPE),^40^ among others.

As can be readily observed from the variety of polymers used in AM and generally in the manufacturing industry, the world is dominated by plastic products. When these products reach their end-of-life cycle and are not recycled, they pollute the environment, causing severe disruptions such as microplastics.^41–43^ Recent studies have provided the first evidence of microplastics in the human placenta,^44^ highlighting the severity of this problem. Most polymers have very long degradation times, ranging from 58 years for bottles to 1200 years for pipes manufactured using HDPE.^45^ These figures correspond to a specific surface degradation rate (SSDR) of 0–11 μm year.^45^ From this perspective, the projection for accumulated plastic waste generation is to reach almost 10.000 million metric tons by 2025, whereas recycling will reach only approximately 2.000 million metric tons in the same year.^46^ To tackle this acute problem and the polluting nature of polymers, the scientific community has investigated biodegradable polymers, which are environmentally friendly^47–49^ and can be removed from the environment faster than other polymers. Nevertheless, the availability of suitable biodegradable materials remains a significant challenge, because most existing biopolymers exhibit limitations in terms of mechanical strength, thermal stability, and printability. Therefore, the scientific community widely investigates and evaluates renewable, sustainable, and biobased polymers in the form of pure materials or composites in the endeavor for cleaner polymeric materials not just in AM but in all fields polymeric materials are used.^50–52^

Along with biodegradable polymers, we also have the ability to utilize naturally occurring polymers such as polyhydroxyalkanoates (PHAs) to address the above-mentioned problems. PHA is a naturally occurring polyester that is involved in the bacterial fermentation of sugars and lipids.^53^ PHAs have emerged as a promising class of microbially produced polyesters and are characterized by their biodegradability, biocompatibility, and versatile material properties.^54–56^ PHAs are thermoplastic biopolymers that have numerous applications, predominantly in the biomedical and food packaging industries, owing^57,58^ to their nontoxicity, biocompatibility, and elastomeric behavior. PHAs are generally characterized by low melting and glass transition temperatures, making them ideal candidates for easy manufacturing processes.^59^ As a result, they have been extensively studied in the past in an effort to elucidate in detail their intriguing physicochemical and mechanical properties.

PHAs can be found either as amorphous or exhibit high crystallinity, such as poly­(3-hydroxybutyrate) and P3HB, which exhibit high brittleness, rendering them less favorable for packaging applications.^60,61^ To enhance the mechanical properties of various polymers, particularly biopolymers, which are important for reducing the negative impact of plastics on the environment, various composites have been prepared based on PHAs.^62^ The important aspect here is the synthesis of a PHA-based composite in which the filler material also exhibits biocompatibility or biodegradability. Such filler materials include biocompatible and biodegradable cellulose nanocrystals (CNCs).^63–66^ CNCs are biobased nanofillers derived from natural cellulose sources that possess high aspect ratios, excellent mechanical strengths, and strong interfacial interactions with polymer matrices.^67,68^ CNCs are usually found in rod or spherical shapes with dimensions ranging from a few to several nanometers.^69^ They are produced from various cellulose-rich natural materials, such as cotton, which contains more than 90% cellulose.^70^ They can also be prepared from recycled cotton or other cellulose-rich materials such as paper or wood to enhance environmental protection through circular economic principles.^71,72^ Owing to their high surface area, hydrogen bonding, and crystallinity modulation capabilities, CNCs have demonstrated significant potential as reinforcing agents for polymer composites.^73,74^

CNCs have been shown to enhance the mechanical properties of PHA nanocomposites,^66^ thus providing additional options for their use as biodegradable and biocompatible PHA/CNS nanocomposites. When introduced into PHA, CNCs are found to increase their crystallization rate as they act as nucleation agents, improve their overall mechanical properties, and constitute PHA/CNC nanocomposites as ideal candidates for biomedical applications such as implants or bioabsorbable/biodegradable sutures and stents.^75,76^

PHA/PLA blends have also recently found applications in AM with the aim of elucidating their mechanical, thermal, and physicochemical properties.^77^ Despite extensive research on the synthesis process, elucidation of the mechanical properties, and overall systematic work performed on biopolymers, very little attention has been paid to the elucidation of the mechanical, structural, rheological, and physicochemical characteristics and properties of PHA/CNC nanobiocomposites, particularly using AM.

AM has proven to be a versatile technique that provides fast manufacturing of complex-shaped materials, along with a significant reduction in material waste when compared with other traditional manufacturing techniques. The latter constitutes AM as a competitive technique, particularly when considering the economic impact and cost of manufacturing.^78^ Therefore, when we combine natural-based polymers that have a minimal impact on the environment, such as PHAs, along with other biological nanocomposites, with the aim of enhancing the mechanical and overall physicochemical properties of the corresponding materials, such as CNCs, we have a manufacturing protocol that can prove to be very important for a sustainable and viable future.

The integration of polyhydroxyalkanoates (PHA) and cellulose nanocrystals (CNCs) in material extrusion (MEX) 3D printing represents a novel advancement in sustainable additive manufacturing. This approach is advantageous as it employs a sustainable method to produce a nanocomposite using two fully biobased and biodegradable materials, addressing the challenges associated with petroleum-based polymers while enhancing mechanical and functional properties. The incorporation of CNCs into PHA offers a promising strategy to improve the thermal stability, stiffness, and dimensional accuracy of 3D printed components, effectively mitigating issues inherent in neat PHA systems. Furthermore, CNCs function as nucleating and rheological modifying agents, enhancing printability and interlayer adhesion of extruded layers. From a processing perspective, the relatively low melting temperature of PHA compared to other common plastics facilitates energy-efficient printing and supports decentralized or small-scale production, owing to the reinforcement and stabilization provided by CNCs. Additionally, sourcing both PHA and CNCs from renewable or waste biomass aligns with circular economy principles, offering a systemic approach that complements material innovation with enhanced environmental stewardship. Consequently, the utilization of PHA/CNC nanocomposites in MEX 3D printing represents a unique convergence of green chemistry, nanotechnology, and advanced manufacturing, heralding an emerging field in the development of next-generation biobased materials.

In this study, we aimed to systematically follow the effect of the addition of CNCs to the PHA polymeric matrix and elucidate the optimum synthetic protocol to maximize the mechanical properties of the corresponding PHA/CNC nanobiocomposites within the context of MEX AM. Correspondingly, we prepared a series of PHA/CNC nanobiocomposites, where the CNC content was gradually increased in intervals of 0.5 wt % CNC. For comparison, we started with pure PHA, which served as a control sample. The other PHA/CNC nanobiocomposites are PHA/0.5%CNC nanobiocomposites (nbc), PHA/1.0%CNC nbc, PHA/1.5%CNC nbc, PHA/2.0%CNC nbc, and PHA/2.5%CNC nbc. All percentages are in wt % CNC.

Raman spectroscopy was employed to closely monitor any structural changes that could be linked to the overall mechanical and physicochemical properties of the corresponding PHA/CNC nanocomposites. To integrate our findings and create a holistic picture of the properties of PHA/CNC nanobiocomposites, we used differential scanning calorimetry (DSC), scanning electron microscopy (SEM), dynamic mechanical analysis (DMA), rheological investigation, thermogravimetric analysis (TGA), and microcomputed high-resolution tomography (μ-CT). To our knowledge, there is a significant lack of studies that deal specifically with the elucidation of a broad spectrum of properties of PHA/CNC nanobiocomposites, and even fewer deal with the direct application of relevant materials through AM processes. Our work aims to shed light on the preparation of PHA/CNCs through AM, while enhancing their mechanical properties, which could lead to the increased use of biodegradable biopolymers as substitutes for other polymers that pose significant threats to environmental safety. The main challenge was to prepare nanocomposites with both matrix and filler eco-friendly, nature-sourced materials and achieve enhanced mechanical performance and processability in MEX AM while maintaining or improving the remaining properties (thermal and structural integrity, 3D printing structure, and rheology). The ability to use biopolymers in applications requiring enhanced mechanical strength can revolutionize the plastic manufacturing industry and lead to a more sustainable future.

## 2 Materials and Methods

The experimental protocol that was followed to prepare the various specimens and evaluate their mechanical, structural, rheological, morphological, and thermal properties is shown in Figure 1. Figure 1a,b shows the starting pure biomaterials, PHA and CNC, after they were dried and weighed to determine the appropriate properties for synthesizing various PHA/CNC nanobiocomposites. Figure 1c,e shows the filament production process through melt extrusion (MEX), drying, and subsequent mechanical testing. Following this stage of the experimental protocol, the MEX AM process follows, as shown in Figure 1f. Finally, the last segment of the experimental protocol, namely the experimental process and subsequent assessment of the various properties of the PHA/CNC nanobiocomposites, are shown in Figure 1g–l. These figures depict the various mechanical tests and elemental, rheological, and thermal evaluations of the various specimens, followed by morphological characterization using SEM.

**Figure 1:**
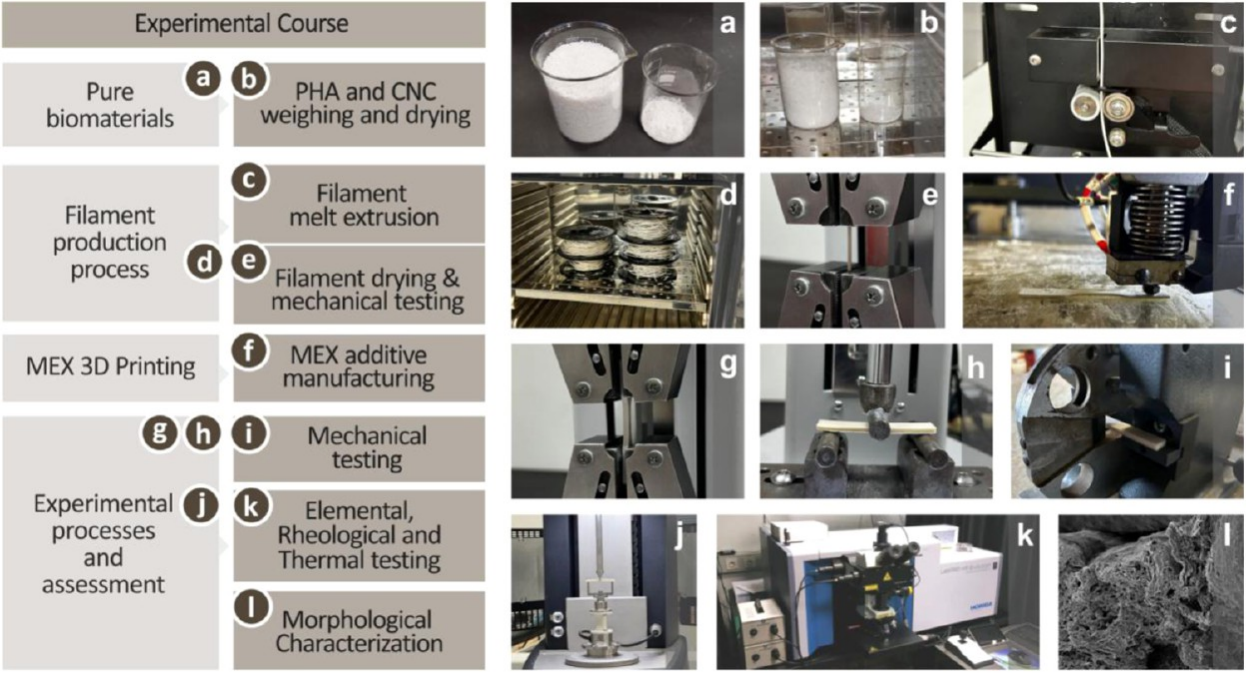
(a, b) Starting materials, PHA and CNC, weighing and drying, (c–e) filament extrusion process and subsequent mechanical testing, (f) MEX AM process, (g–l) series of extensive experimental assessments of the various PHA/CNC nanobiocomposites.

### 2.1 Reagent Materials

To synthesize the various PHA/CNC nanobiocomposites investigated in this study, we utilized the following starting materials: PHA was acquired from ColorFabb (Belfeld, NL) in the form of pellets, bearing the commercial name AllPHA ColorFabb. The manufacturer does not disclose the exact composition of the product. According to its safety data sheet, the formulation contains less than 2.5% titanium dioxide. The product is classified as nonflammable and does not meet the criteria for PBT (persistent, bioaccumulative, and toxic) or vPvB (very persistent and very bioaccumulative) substances. Additionally, it is not considered an environmental hazard. Certification from TUV Austria Belgium confirms that the product satisfies the 90% biodegradation threshold.

The manufacturer states that ″allPHA″ is produced through fermentation, a natural biochemical process whereby bacteria are supplied with natural sugars and oils to produce polyhydroxyalkanoates (PHAs), which serve as ″fat″ cells. This material is designed to biodegrade in any environment without generating microplastics. When bacteria metabolize substrates such as glucose, sucrose, vegetable oils, or fatty acids, they synthesize various PHAs—biodegradable polyesters functioning as intracellular carbon and energy reserves. The specific type of PHA generated depends on the carbon source and bacterial strain employed.

Sugars like glucose and sucrose typically lead to the production of polyhydroxybutyrate (PHB) or its copolymer PHB-*co*-HV, both classified as short-chain-length PHAs (scl-PHAs), commonly synthesized by bacteria such as Cupriavidus necator. PHB is a brittle thermoplastic similar to polypropylene. In contrast, medium-chain-length PHAs (mcl-PHAs), such as those derived from vegetable oil or waste cooking oil, are synthesized by bacteria like *Pseudomonas putida* and *Pseudomonas aeruginosa* and exhibit more elastomeric and flexible properties, making them suitable for medical and packaging applications. The ″allPHA″ material under discussion is a PHA, rather than a PHB; however, further details have not been provided by the manufacturer. This study specifically examines the ″allPHA″ commercial PHA product, and thus, results should be interpreted as applicable only to this grade, as outcomes may differ with other PHA grades or types.

CNCs were acquired from Nanografi Nanotechnology (Ankara, TR) in a nanopowder form. The technical specifications according to the manufacturer datasheet are presented in the following Table 1.

**Table 1:** CNC Nanoparticles Technical Specifications, According to the Nanographi Datasheet

average particle size	10–20 nm wide, 300–900 nm length
form	spray dried powder (<6.0% moisture)
cellulose crystallinity (XRD)	92%
density	1.49 g/cm^3^
bulk density	0.5–0.8 g/cm^3^
decomposition temperature of crystalline nanocellulose (TGA in N_2_)	349 °C
pH, dispersed powder at 2% (w/w)	5.0–8.0
particle size, dispersed powder at 2% (w/w)	<150 nm
viscosity, dispersed powder at 2% (w/w)	>5 cP
conductivity, dispersed powder at 2% (w/w)	<350 μS/cm

The starting materials, as shown in Figure 1a,b, were dried overnight to remove any absorbed H_2_O from the atmosphere.

### 2.2 Initial Materials Preparation and Filament Production

Immediately after drying and removing the starting raw materials from the oven, as stated above, we carefully weighed each component corresponding to the binary mixtures of PHA and CNC, and then proceeded to extensive mixing to produce different PHA/CNC nanobiocomposites. The final batches were PHA pure, to serve as a control specimen to which all other PHA/CNC nanobiocomposites would be compared and PHA/CNC nanobiocomposites bearing 0.5, 1.0, 1.5, 2.0, and 2.5 wt % CNC quantities into the PHA polymer matrix. These quantities were carefully chosen after performing the initial experiments, which showed that no significant changes were observed in any of the properties tested beyond the 2.5 wt % loading. These are discussed in detail later in this paper. This has also been observed in previous studies regarding the synthesis of PHA/CNC nanobiocomposites for CNC quantities far above ∼2 wt % and significant nanoparticle agglomeration was observed, leading to the overall degradation of the final PHA/CNC nanobiocomposite during processing (not in 3D printing).^64^ The final PHA/CNC nanobiocomposite batches were subsequently fed into a filament extruder, which simultaneously performed mixing and filament production for the MEX 3D printing. The filament produced had a nominal diameter of 1.75 mm which is the standard for 3D printing (±0.1 mm deviation was achieved, which is acceptable for MEX AM). The extruder features a screw that was developed with special geometric characteristics (3Devo Composer 450, The Netherlands) to ensure optimal mixing of the feeder material for filament production, achieving excellent dispersion of the binary mixture and its additives, as confirmed by extensive testing (which also established the extrusion settings for all compounds for reliable comparison), as shown in the Supporting Information along with this work.

### 2.3 3D Printing of the Various PHA/CNC Nanobiocomposites

After the production of high-quality filaments corresponding to the various PHA/CNC nanobiocomposites, as described above, we utilized a Funmat HT 3D printer from the company Intamsys Technology Co. Ltd., established in Shanghai, CN, operating in MEX mode to prepare 3D printed samples corresponding to the various CNC loading PHA/CNC nanobiocomposites. Nozzle (0.4 mm in diameter) temperature was adjusted at 200 °C, while the build plate temperature was set at 40 °C. A 0° orientation and ± 45° rectilinear infill was used. Printing used 2 perimeters, no top or bottom solid layers, a speed of 50 mm/s and 0.2 mm layer height. For each PHA/CNC nanobiocomposite, five different specimens were 3D printed (per mechanical test) in accordance with the respective standards (please see the Supporting Information) to perform various mechanical, rheological, structural, and physicochemical analyses.

### 2.4 Characterization of PHA/CNC Nanobiocomposites through Vibrational Spectroscopy, Rheology, and Thermal Properties Evaluation

To account for the structural characteristics of the various PHA/CNC nanobiocomposites prepared via 3D printing, we utilized a LabRAM HR Raman spectrometer (HORIBA, Kyoto, JP). A Rotational rheometer (Hybrid) model DHR-20 Discovery (TA Instruments, Delaware) was used to assess the rheological behavior of the various PHA/CNC nanobiocomposites. Their thermal properties were examined using a Discovery Simultaneous Thermal Analyzer SDT 650 (TA Instruments, Delaware) and a DSC 25 (TA Instruments). The methodology used for each of these characterization methods is presented in the Supporting Information.

### 2.5 Evaluation of the Mechanical Properties of the PHA/CNC Nanobiocomposites

One of the most intriguing aspects of 3D-printed PHA/CNC nanobiocomposites is the elucidation of their mechanical properties and how they are influenced by CNC content. For this purpose, we utilized an MX2 motorized test stand by Imada Inc. (Tokyo, JP) and performed a series of tensile and bending experiments, starting from filaments corresponding to various PHA/CNC nanocomposites. The specimens were positioned in special grips on MX2, and the testing was performed according to the appropriate American Society for Testing and Materials (ASTM). In this regard, we utilized the ASTM D638-14 and ASTM D790-10 standards, employing a 10 mm/min elongation speed on the samples under evaluation. Accounting for impact testing (Charpy notched), we used an MT220 instrument acquired by Terco AB (Kungens, SE) following the ASTM D6110 standard, which dictates the release height for the hammer to be set at 367 mm. Finally, we evaluated the Vickers microhardness of the various specimens using a Test 300 Vickers instrument acquired by Innovatest Europe BV (Maastricht, NL), following the ASTM E384-17 standard.

Finally, we carried out DMA analysis using a Discovery Hybrid Rheometer DHR20 equipped with a 3-point bending setup with a 40 mm span. The dimensions of the test specimens were 10 mm Ã–50 mm Ã–3 mm. The temperature range for conducting the experiments was between 30 and 220 °C with a temperature gradient of 5 °C/min. During the entire experimental process, the samples were subjected to sinusoidal strains between 0 and 0.05% at a frequency of 1.0 Hz. For consistency and to maintain full contact with the specimen throughout the process, we employed a force-tracking mechanism.

### 2.6 Morphological Characterization of the PHA/CNC Nanobiocomposites through HD μ-CT and SEM

To elucidate the influence of the addition of CNC to the PHA polymer matrix and the subsequent 3D printed samples and their morphology, for every PHA/CNC nanobiocomposite, we carried out high-definition microcomputed tomography using an HV compact Tomoscope from Werth (Berlin, DE). We evaluated the geometrical characteristics and their deviations from nominal values, along with the voids present after the 3D printing of the structure (porosity). A configuration with a resolution of 72.58 μm (75 L) along the *X*-axis and 72.65 μm along the *Y*-axis was employed to assess dimensional accuracy. A configuration with a resolution of 15.46 μm (16 L) on the *X*-axis and 15.49 μm on the *Y*-axis was utilized for evaluating porosity. Both configurations comprised 1600 sections per revolution.

A model named JSM-IT700HR SEM (the type of the apparatus was field-emission) (Jeol, Tokyo, JP) was employed to acquire detailed images of the fractured surfaces produced during mechanical testing to evaluate the fracture mechanism, as well as the side surface of the samples to assess the overall quality of the 3D printed PHA/CNC nanobiocomposites (20 kV, gold-sputtered samples, high vacuum mode).

## 3 Results

### 3.1 Results from Vibrational Spectroscopy

From all the 3D printed specimens, namely pure PHA and the various PHA/CNC nanobiocomposites, we acquired high-quality Raman spectra, which are shown in Figure 2. Figure 2a depicts the Raman spectra of pure and stacked PHA/CNC nanocomposites with CNC concentrations ranging from 0.5 to 2.5 wt % in 0.5 wt % intervals. Figure 2b shows the Raman spectra of the various PHA/CNC nanobiocomposites after subtracting the pure Raman spectra of PHA. This allows for direct observation of the structural alterations depicted in the various Raman spectra and is attributed to the CNC filler content variation. Table 2 summarizes the various Raman-active peaks originating from pure PHA polymers established in the relevant literature.

**Figure 2:**
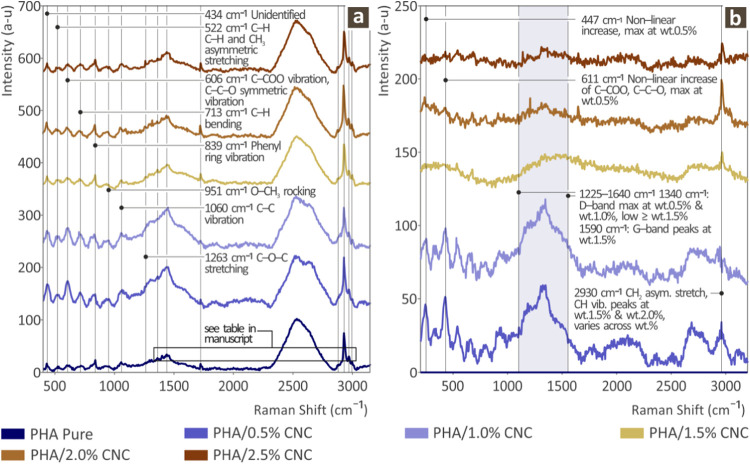
Raman spectra from PHA pure and PHA/CNC (0.5, 1.0, 1.5, 2.0, 2.5 wt %) nanobiocomposites. (a) Raman spectra of all prepared samples and (b) Raman spectra after subtracting the PHA pure from all PHA/CNC nanobiocomposites.

**Table 2:** Significant Raman Active Peaks of PHA Pure and Their Assignment to Structural Units Present

wavenumber (cm^–1^)	intensity	Raman peak assignment
434	medium	unidentified
522	small	C–H and CH_3_ asymmetric stretching^79^
606	medium	C–COO vibration, C–C–O symmetric vibration^79^
713	small	C–H bending^80^
839	medium	phenyl ring vibration^80,81^
951	small	O–CH_3_ rocking^79^
1060	medium	C–C vibration^82^
1263	small	C–O–C stretching^79^
1364	small	C–C–H, C–O–H, and O–C–H^83^
1440	small	CH_3_ asymmetric stretching^84^
1725	medium	C=O bond stretching;^79,85^ C–O–C symmetric stretching^86^
2880	small	CH_2_, CH_3_ vibration^82^
2930	very strong	CH_2_ asymmetric stretching; CH vibration^87^
2970	strong	C–H stretching^81^
2996	medium	C–H stretching^81^

As depicted in Figure 2b, the addition of CNC to the PHA polymer matrix resulted in multiple changes in the Raman intensity. In the Raman lines (447 and 611 cm^–1^) the Raman signal intensity presented a nonlinear increase with the highest intensity at 0.5 wt % CNC loading and decreasing as the CNC wt % grows. The Raman spectral band between 1225 and 1640 cm^–1^ also exhibited a nonlinear behavior. The graphite band (D-band) at 1340 cm^–1^ appeared strongly in the samples 0.5 and 1.0 wt %, while the graphite band (G-band) at 1590 cm^–1^ appeared mostly in the sample 1.5 wt %. In the samples 1.5, 2.0, and 2.5 wt % D-band was low. Finally, a strong increase in CH_2_ asymmetric stretching; CH vibration was present, while inconsistent between the different wt %, presented a maximum on the 1.5 and 2.0 wt % CNC. All changes presented with their related assignments are listed in Table 3.

**Table 3:** Observed Variations Among the Raman Active Peaks for PHA Pure and PHA/CNC Nanobiocomposites

447	nonlinear increase	a nonlinear increase of the Raman peak intensity appeared with the highest intensity at 0.5 wt % and decreased as the wt % increased.
611	nonlinear increase	a nonlinear increase of C–COO vibration, C–C–O symmetric vibration appeared, with the highest intensity at 0.5 wt % and decreasing as the wt % is increasing.
1225–1640	nonlinear increase	a broad nonlinear increase of the Raman spectrum. The D-band at 1340 cm^–1^ appears strongly in the samples 0.5 and 1.0 wt %, while the G-band at 1590 cm^–1^ appears mostly in sample 1.5 wt %. in samples 1.5, 2.0, and 2.5 wt % D-band is low.
2930	increase	a strong increase of CH_2_ asymmetric stretching; and CH vibration, while inconsistent between the different wt % shows a maximum on the 1.5 and 2.0 wt %, respectively.

### 3.2 Rheological and Thermal Characteristics of the PHA/CNC Nanobiocomposites

The rheological characteristics of the PHA/CNC nanocomposites and pure PHA are shown in Figure 3. Figure 3a shows the stress and viscosity versus the shear rate at 190 °C behavior of various specimens. These results imply that with increasing stress rate the viscosity decreases while Figure 3b shows the melt flow rate (MFR) at 190 °C which indicates that as the CNC content increases the MFR decreases, exhibiting a small fluctuation around 1.5 wt % CNC loading. Overall, the maximum MFR was observed for the pure PHA polymers.

**Figure 3:**
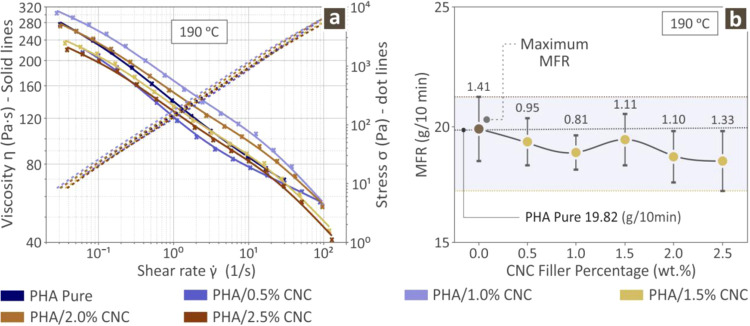
Rheology results for the various PHA/CNC nanobiocomposites prepared and PHA pure (a) stress and viscosity versus shear rate results and (b) MFR per CNC filler percentage (average values and deviation presented with error bars, calculated from the samples tested per case).

The thermal characteristics of the pure PHA and various PHA/CNC nanobiocomposites are shown in Figure 4. Figure 4a shows the TGA curves. The final residue (FR) and initial decomposition temperature (IDT) are shown in Figure 4d. The IDT values were maximum for pure PHA (with small variation, i.e., decrease, in the compounds), whereas the FR values were maximum for the highest loading of the PHA/CNC nanobiocomposite, as expected. Figure 4b shows the DSC graphs for the heat flow versus temperature, depicting the melting temperature *T*_m_ for all prepared samples. These measurements are shown in Figure 4c. It is readily shown that *T*_m_ has a very small fluctuation with varying CNC content, and the minimum *T*_m_ from the maximum *T*_m_ differs only by 0.8 °C.

**Figure 4:**
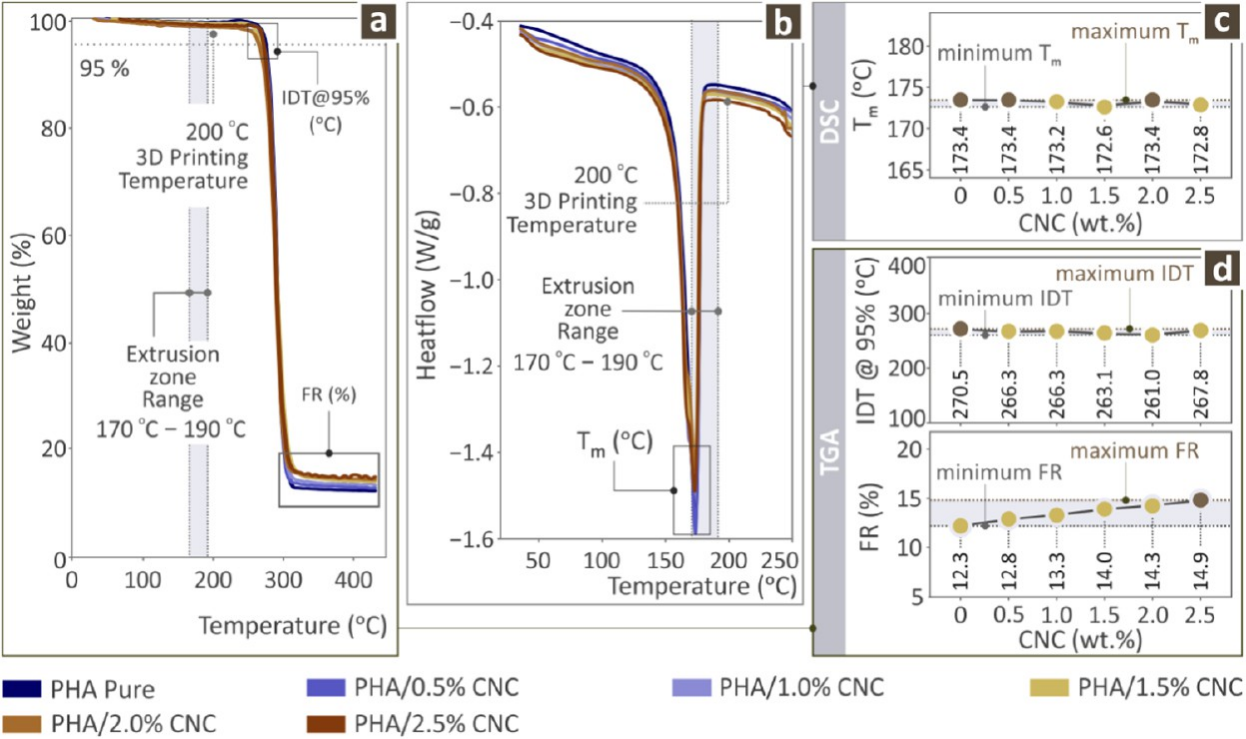
Thermal properties graphical representation of the unfilled PHA and PHA/CNC nanobiocomposite samples through (a) TGA and (b) DSC graphs, as well as their respective (c) *T*_m_, (d) FR, and IDT values.

### 3.3 Evaluating the Mechanical Properties of the PHA/CNC Nanobiocomposites

The DMA graphs acquired from all the evaluated samples are shown in Figure 5. Figure 5a–f shows the loss and storage modulus along with tan­(δ) vs temperature, starting from pure PHA (Figure 5a) and all PHA/CNC nanobiocomposites (Figure 5b–5f). As shown in Figure 5, as the storage and loss moduli decreased, tan­(δ) increased until it reached the melting temperature of each specimen, after which it started to decrease abruptly.

**Figure 5:**
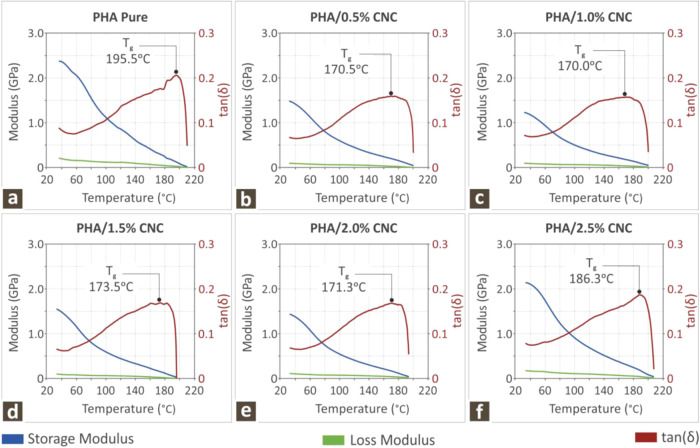
DMA curved depicting the storage modulus, loss modulus, and tan­(δ) compared to temperature for (a–f) PHA pure and PHA/CNC nanobiocomposites, respectively.

The next step was to assess the tensile properties of the PHA/CNC nanocomposites. Figure 6 shows the results acquired from tensile testing of pure PHA and various PHA/CNC nanobiocomposites. Figure 6a depicts the tensile stress values acquired for various specimens, and the inset photo shows a randomly selected sample placed during the experimental process. Tensile testing revealed that the values increased with increasing CNC content and were maximized for the PHA/1.5 wt %CNC nanobiocomposite, showing an improvement with respect to pure PHA by 8.6%. Interestingly, as the CNC filler content increased, the tensile stress values deteriorated significantly, thereby minimizing the highest CNC filler content in the nanobiocomposites.

**Figure 6:**
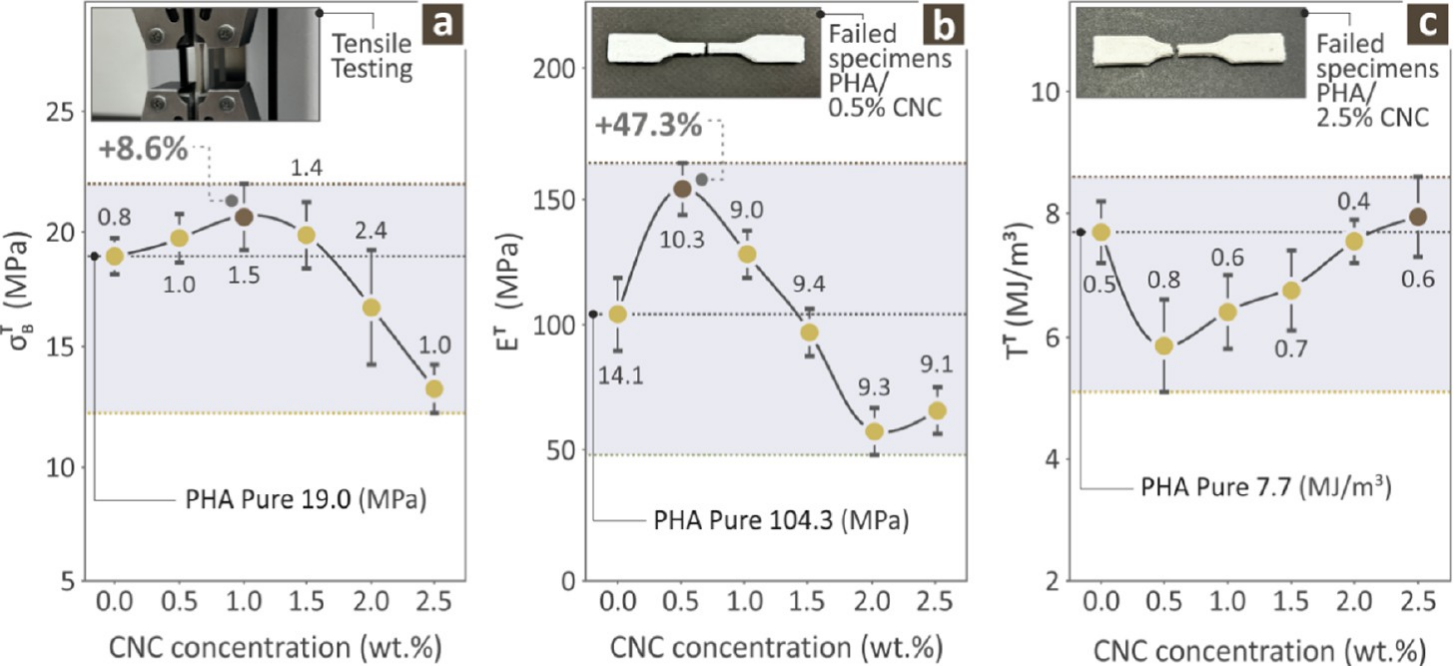
Tensile stress values originating from the various PHA pure and PHA/CNC nanobiocomposites tensile (a) strength, (b) modulus of elasticity, and (c) toughness (average values and deviation presented with error bars, calculated from the samples tested per case).

Figure 6b shows the Young’s modulus and an inset photo of a failed specimen from the PHA/0.5 wt % CNC nanobiocomposite. The values for Young’s modulus show somewhat different behavior with increasing CNC filler content, as are maximized for the 0.5 wt % CNC filler percentage, showing a remarkable 47.3% increase with respect to pure PHA; as the CNC additive content was increased, the values decreased steadily, minimizing for the 2.0 wt % CNC filler percentage PHA/CNC nanobiocomposite, and for the highest content PHA/CNC nanobiocomposite shows a small, almost negligible increase. Finally, Figure 6c shows the tensile toughness of the various PHA/CNC nanobiocomposites with an insert photo of a failed PHA/2.5 wt % CNC nanobiocomposite. Interestingly, as the CNC filler percentage steadily increased in the PHA/CNC nanobiocomposites, the value dropped abruptly for the PHA/0.5 wt % CNC nanobiocomposite, and as the CNC content continued to increase, the values seemed to increase steadily, maximizing the highest CNC filler content, which was almost the same as that of the PHA pure polymer.

Figure 7 illustrates the outcome of the bending experiments performed on the unfilled PHA and the PHA/CNC nanobiocomposites. Figure 7a depicts the bending strength values, along with an inset photo showing the experimental bending process for a randomly selected specimen. Figure 7a shows that as the CNC content increased, the bending strength increased rapidly, maximizing the PHA/0.5 wt % CNC nanobiocomposite by 23.3% with respect to pure PHA. A further increase in the CNC filler content led to a gradual decrease in the bending strength values, which seemed to be minimized for the PHA/2.0 wt % CNC nanobiocomposite, while the maximization of the CNC filler percentage led to a small increase in the bending strength of the PHA/CNC nanobiocomposite. Figure 7b shows the bending modulus of elasticity along with an inset photo depicting the tested specimen of the PHA/0.5 wt % CNC nanobiocomposite. Again, with increasing CNC filler percentage, a rapid increase in the bending modulus of elasticity was observed, maximizing for the PHA/0.5 wt % CNC nanobiocomposite exhibiting an increase of 20.8% with respect to the PHA pure polymer. A further increase in the CNC filler content led to a steady decrease in the bending modulus of elasticity, which was minimized for the PHA/CNC nanobiocomposite with the highest CNC filler content. Finally, Figure 7c shows the bending toughness values for the pure PHA and the various PHA/CNC nanobiocomposites, along with an inset photo of a tested specimen, namely the PHA/2.5 wt % CNC nanobiocomposite. As the CNC filler content increased, the bending toughness decreased rapidly and reached its minimum value for the PHA/0.5 wt % CNC nanobiocomposite. With further increase of the CNC filler percentage the values of the bending toughness steadily increase and are maximized for the highest CNC filler percentage, namely the PHA/2.5 wt % CNC nanobiocomposite, leading to a 9.5% increase with the values acquired for the PHA pure specimen.

**Figure 7:**
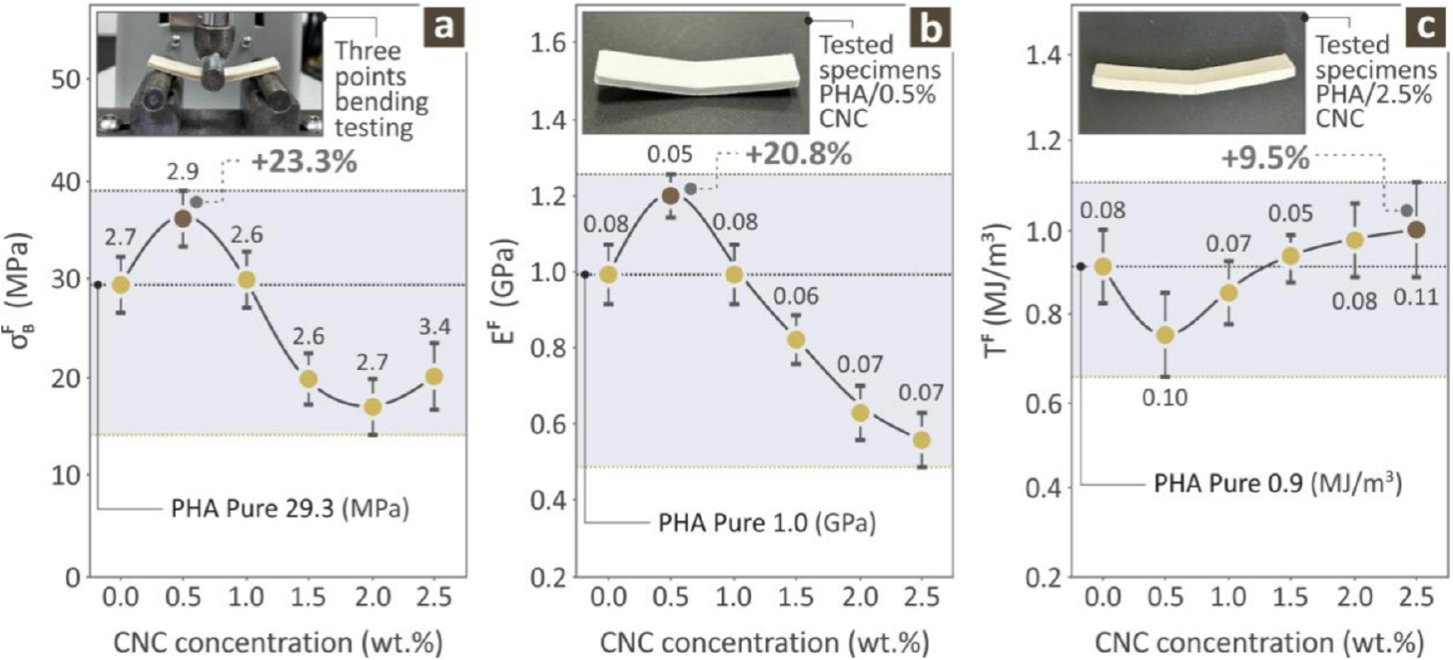
Bending toughness values acquired from the PHA pure and the various PHA/CNC nanobiocomposite specimens; (a) bending strength and an illustration from the bending experiment of a randomly selected example, (b) bending modulus of elasticity and an illustration from a PHA/0.5 wt % CNC nanobiocomposite specimen, (c) bending toughness and an image from a PHA/2.5 wt % CNC nanobiocomposite specimen (average values and deviation presented with error bars, calculated from the samples tested per case).

Figure 8a shows the corresponding values for the tensile toughness of pure PHA and various PHA/CNC nanobiocomposite filaments, along with an inset photo that shows the experimental process for a randomly selected sample. With increasing CNC filler content, the tensile toughness of the filament decreased significantly, and as the CNC filler content continued to increase, this trend reversed and increased slowly. However, even for the highest CNC filler content, it remained below the value obtained for pure PHA, which was the highest. Figure 8b shows the Charpy impact strengths of pure PHA and various PHA/CNC nanobiocomposites, along with the inset of a failed Charpy specimen from pure PHA. It is readily observed that with increasing CNC filler content, the Charpy impact strength values diminish steadily, minimizing for the highest CNC content PHA/CNC nanobiocomposite, resulting in the pure PHA having the highest Charpy impact strength. Finally, Figure 8c shows the Vickers microhardness values for pure PHA and various PHA/CNC nanobiocomposites, along with an inset photo showing the Vickers imprint on a pure PHA specimen. The same trend was observed for the Vickers microhardness values with increasing CNC filler percentage, that is, the highest values were observed for the PHA pure specimen.

**Figure 8:**
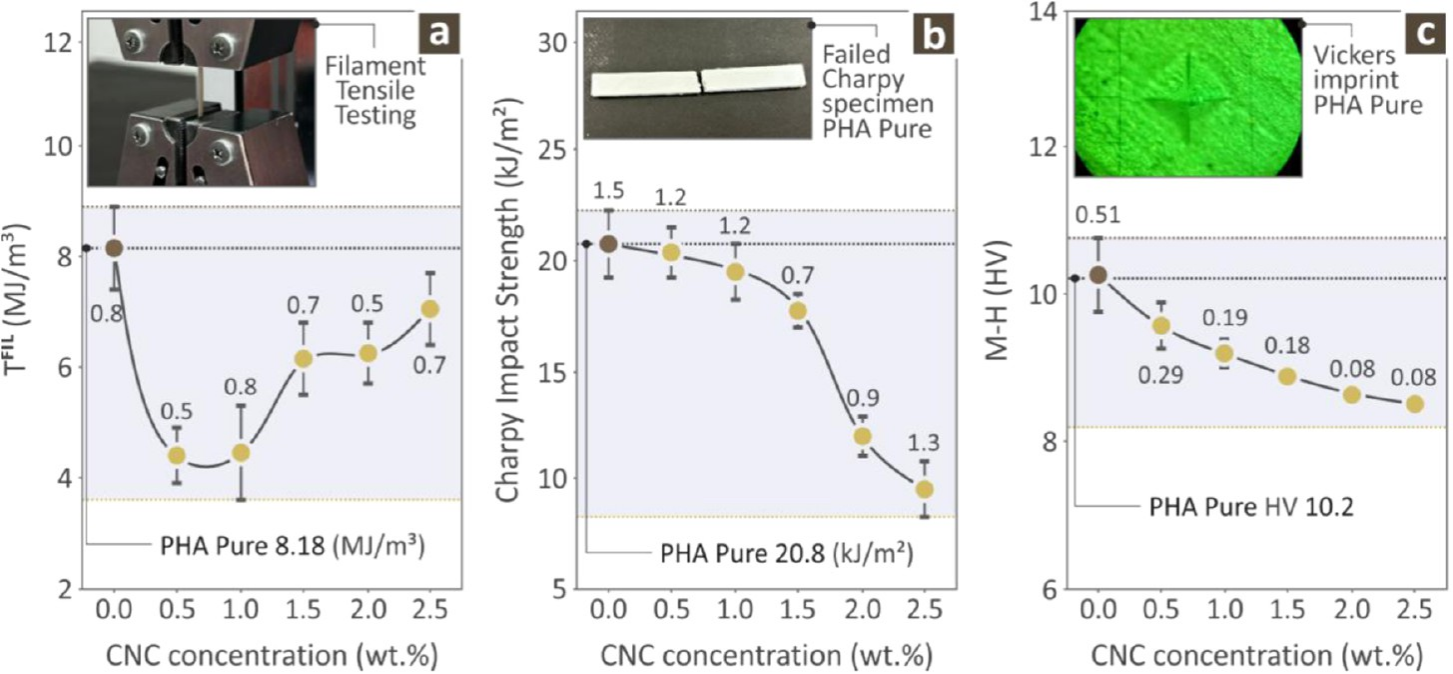
(a) Tensile toughness of all PHA pure and PHA/CNC nanobiocomposite extruded filaments, (b) Charpy impact strength of all PHA pure and PHA/CNC nanobiocomposites, and (c) Vickers microhardness of all PHA pure and PHA/CNC nanobiocomposites (average values and deviation presented with error bars, calculated from the samples tested per case).

### 3.4 Dimensional Analysis and Evaluation of the PHA/CNC Nanobiocomposites

To account for possible alterations or deviations in the dimensions of the 3D printed PHA/CNC nanobiocomposites from the nominal dimensions fed into the 3D printer using a design software, we performed detailed high-definition microcomputed tomography (HD μ-CT). Figure 9 shows the relevant results obtained for all the specimens. Figure 9a,b depicts the dimensional deviation of the PHA/1.0 wt % CNC nanobiocomposite by utilizing a color-coded spectrum. Figure 9c shows the actual-to-nominal (A2N) geometrical characteristics of pure PHA and various PHA/CNC nanobiocomposites prepared at a 95% deviation level. As the CNC particle content increased, the dimensional deviation decreased, reaching a minimum for the PHA/1.0 wt % CNC nanobiocomposite, which is 18.4% lower than that of pure PHA (parts are 3D printed, achieving better geometrical accuracy). A further increase in the CNC filler content led to an increase in the dimensional deviation, which surpassed that of pure PHA for the highest loading of the PHA/CNC nanobiocomposite. Figure 9d,e shows the porosity levels of the PHA/1.0 wt % CNC nanobiocomposite, again using a color-coded spectrum.

**Figure 9:**
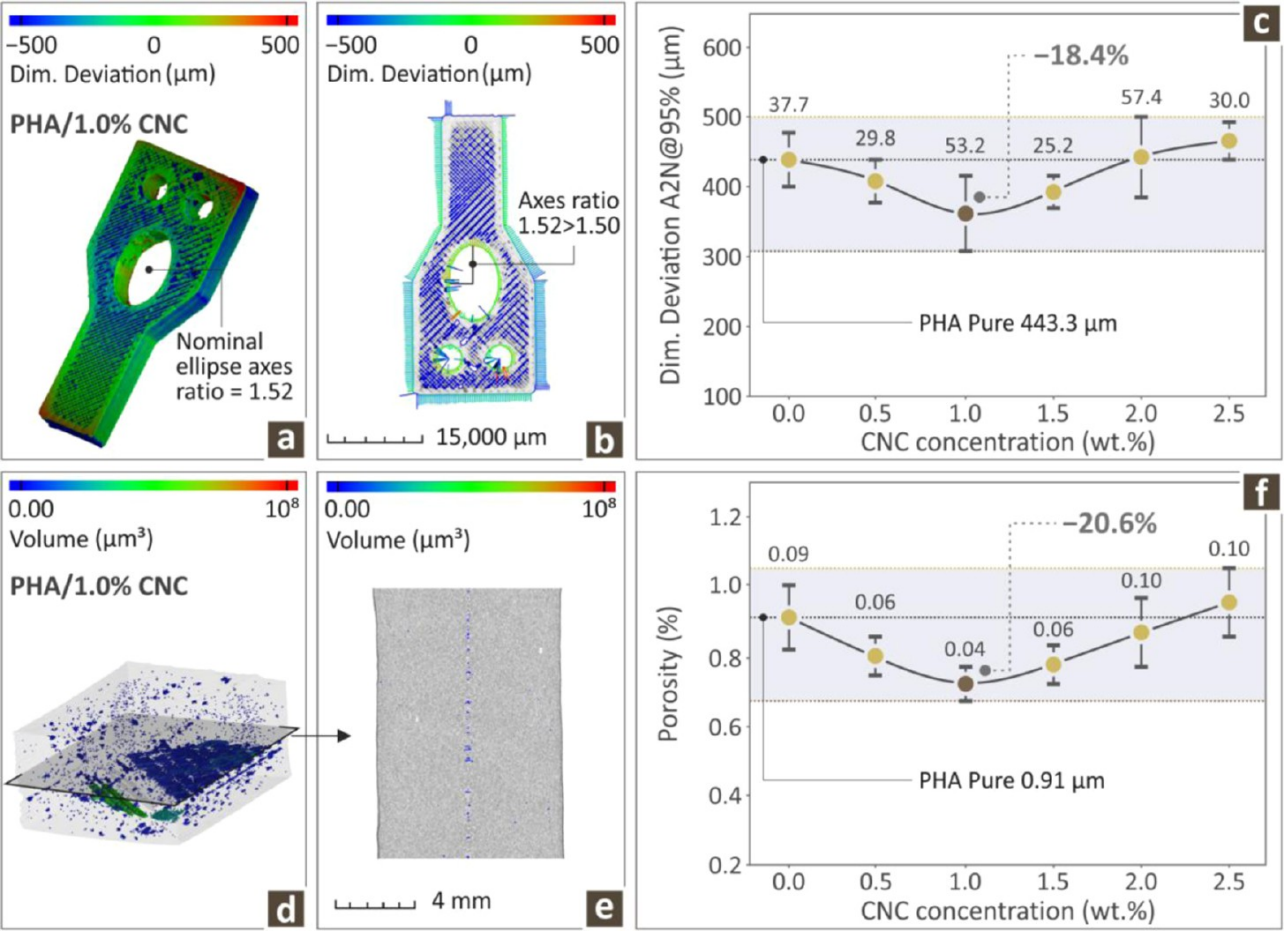
(a, b) Dimensional deviation of a PHA/1.0 wt % CNC bionanocomposite through color-coded mapping, (c) geometrical accuracy values of all PHA/CNC nanocomposites prepared, (d, e) PHA/1.0 wt % CNC section through color-coded mapping, (f) porosity values of all PHA/CNC nanobiocomposites (average values and deviation presented with error bars, calculated from the samples tested per case).

Figure 9f shows the porosity levels as a percentage of the CNC filler content for the pure PHA and various PHA/CNC nanobiocomposites. The incorporation of CNC into the PHA pure polymer matrix leads to a significant decrease in porosity reaching a minimum for the PHA/1.0 wt % CNC nanobiocomposite, exhibiting a value that is 20.6% less than PHA pure. Further increase in the CNC filler content reversed this trend and led to an increase in the number of observed pores.

### 3.5 Morphological Characterization of the PHA/CNC Nanobiocomposites through SEM

Figure 10 shows detailed SEM images acquired from selected PHA/CNC nanocomposites that cover all compositional ranges, from pure PHA to PHA/1.0 wt % CNC, to PHA/2.0 wt % CNC nanobiocomposites, that is, no CNC loading and moderate and high CNC loadings. Figure 10a–c shows SEM images acquired from a PHA pure specimen, at 150× magnification from its side and two subsequent images at 27x and 5000× magnifications from the fractured side (tensile specimens, after they failed in the experiment). Figure 10d–f shows SEM images acquired from a PHA/1.0 wt % CNC nanobiocomposite at the same magnifications and acquisition sides as above, and Figure 10g–I shows SEM images acquired from a PHA/2.0 wt % CNC nanobiocomposite at the same magnifications and acquisition sides. It is readily observed that the addition of CNC favors the overall quality of the moderate-content PHA/CNC nanobiocomposite, in agreement with the results obtained from the various experimental processes described above.

**Figure 10:**
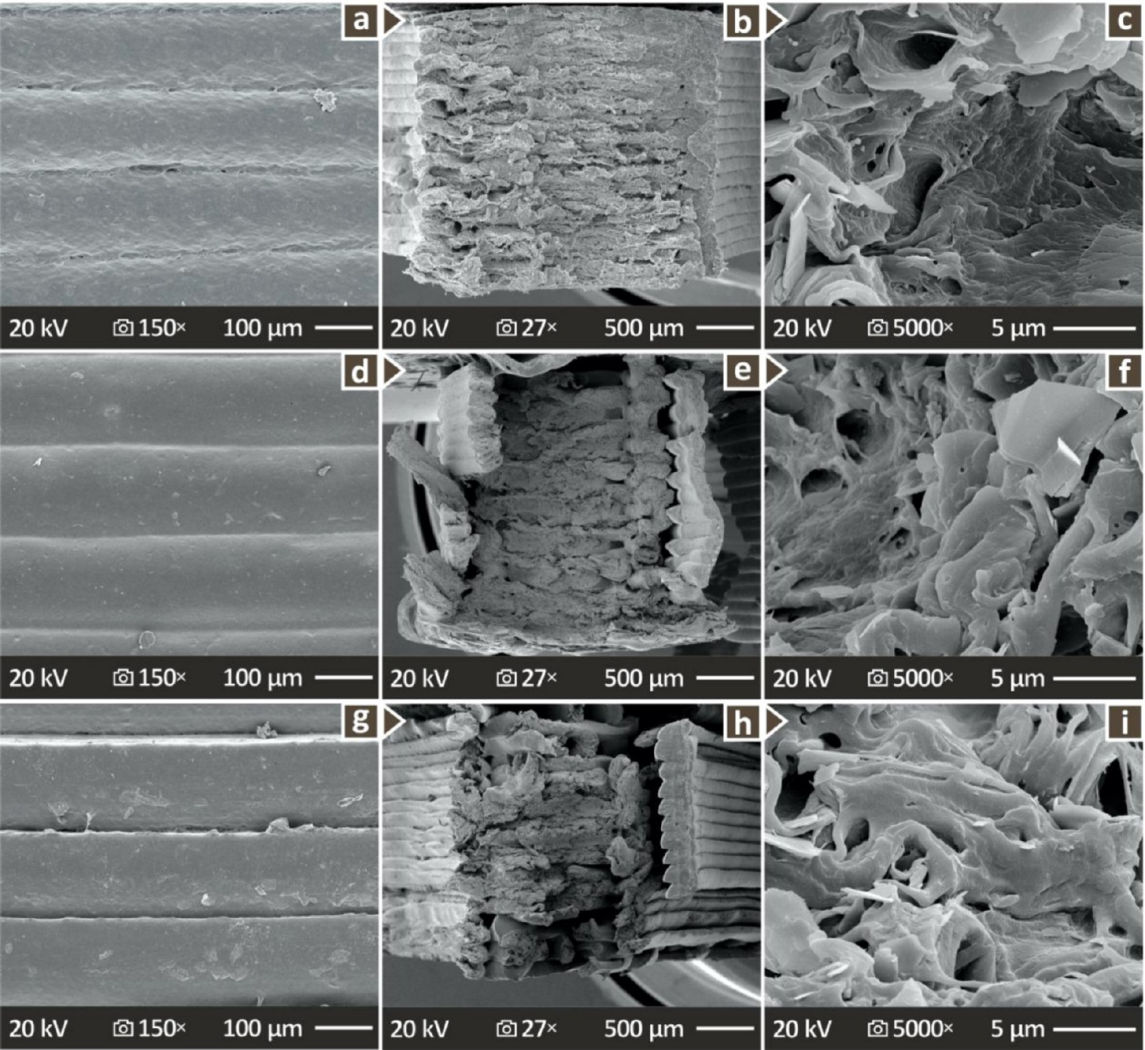
SEM images depicting (a–c) PHA pure, (d–f) PHA/1.0 wt % CNC, and (g–i) PHA/2.0 wt % CNC: lateral surfaces at 150× magnification, and fractured surfaces at 27× and 20,000×.

Figure 11 shows a selected PHA/1.5 wt % CNC nanobiocomposite in more detail, as this compositional range appears to be the most promising in terms of the enhancement of the overall properties examined herein upon the addition of CNC into the PHA pure polymer matrix. Figure 11a–c shows SEM images acquired by a PHA/1.5 wt % CNC nanobiocomposite at 27× magnification, taken from its side, at 150× magnification, taken from its side, and at 27× magnification taken from its fracture side, respectively. Figure 11d–f shows SEM images acquired from a PHA/1.5 wt % CNC nanobiocomposite at 300×, 1000×, and 5000× magnifications, all taken from the fracture side. It is readily observed that the improvement in the overall quality of the samples is confirmed for this sample and agrees well with all documented results with a variety of experimental techniques presented herein.

**Figure 11:**
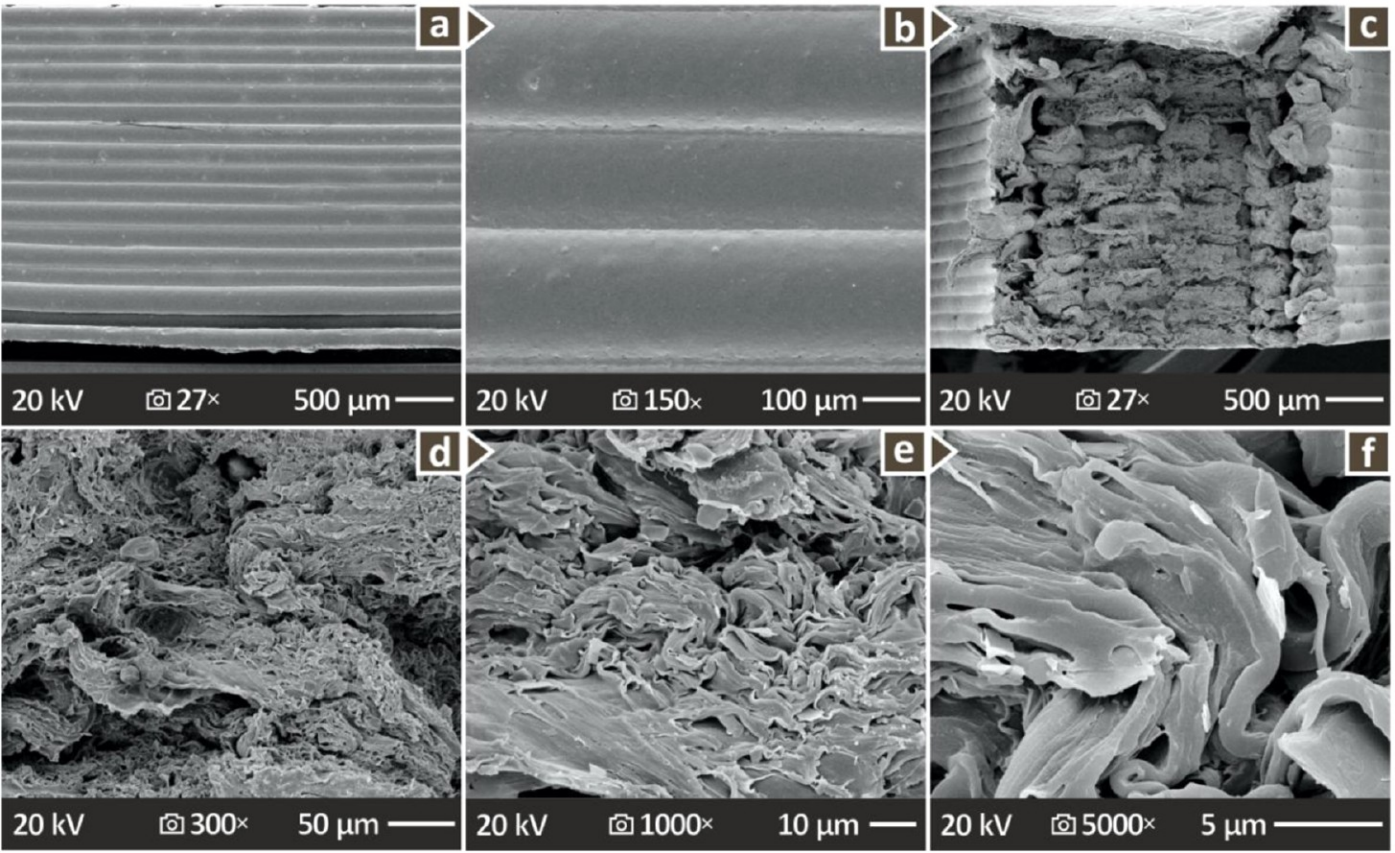
SEM images depicting PHA/1.5 wt % CNC lateral side at (a) 27×, and (b) 150×, and fractured region at (c) 27×, (d) 300× (e) 1000×, and (f) 5000×.

## 4 Discussion

In this study, we analytically investigated the influence of the addition of CNC into the PHA polymer matrix on the mechanical, rheological, thermal, and structural properties of the resulting PHA/CNC nanobiocomposites. For this purpose, we prepared a series of PHA/CNC nanobiocomposites bearing different loadings of CNC, namely 0.5, 1.0, 1.5, 2.0, and 2.5 wt % filler content. For comparison, a pure PHA polymer was prepared as a control sample. As explained previously, the ability to produce PHA/CNC nanobiocomposites via AM is of paramount importance. Combined with multifunctionalities that can positively affect the above-mentioned properties of various PHA/CNC nanobiocomposites, this is a subject that, to the best of our knowledge, remains largely unexplored.

As was readily observed from the Raman spectra acquired from all different PHA/CNC nanobiocomposites, the addition of CNC into the PHA pure polymer matrix altered the vibrational signatures, depicted as broad asymmetric bands at medium and high wavenumbers. For CNC loadings greater than 1.0 wt % CNC, we observed a decrease in the broad asymmetric band between 1100 and 1500 cm^–1^ implying that the structure of the higher-loading PHA/CNC nanobiocomposites became very stiff, and the structural units were even more interconnected, resulting in a very rigid structural network.

This is in agreement with the relevant information acquired from the MFR and the various viscosities and stress behaviors of the various PHA/CNC nanobiocomposites. The MFR appears to be maximum for the PHA pure polymer, whereas it fluctuates as it diminishes with increasing CNC content, reaching a minimum for the highest loading PHA/CNC nanobiocomposite. This must be considered, particularly for AM parts, to adjust the printing parameters accordingly. However, the rheological response of PHA to the addition of CNC did not change significantly. Generally, a high MFR implies better processing of the corresponding material. Therefore, it is expected to have a better mechanical response in the 0.5 wt % compound, which has a rather low viscosity. The improved processability in this case contributed to better mechanical performance. In addition, it led to improved geometrical accuracy and lower porosity in the 3D printing system compared to the unfilled PHA matrix, showing a correlation between the material characterization and quality metrics.

The data presented in Figure 3a demonstrate that the viscosity of all samples decreased as the shear rate increased, exhibiting shear-thinning characteristics. Pure PHA exhibits high initial viscosity with significant shear-thinning behavior, meaning that there is entanglement between the polymer chains, which aligns under shear stresses, leading to easier flow and, hence, lower viscosity. When CNC are introduced, they can disrupt the entanglement of polymer chains and favor interactions between particles rather than between the particles and the polymer matrix. In the case of PHA/0.5% CNC, the viscosity decreased slightly, indicating a small interruption of the polymer chains. CNC can also form interconnected networks or aggregates within the polymer matrix, impeding flow and increasing viscosity.^88,89^ This can be observed in the PHA/1% CNC sample, which presents the highest initial viscosity owing to the aforementioned phenomena. As the CNC content increased, PHA/1.5% CNC and PHA/2.0% CNC exhibited a steady decrease in viscosity, probably owing to the sufficient dispersion of the CNC in the polymer blend, which did not form agglomerates and impeded the flow. Finally, a slight increase in the viscosity of PHA/2.5% CNC was observed, which could be attributed to the elevated percentage of the filler.

The MFR of pure PHA served as the baseline for comparison with all composite samples, as shown in Figure 3b. Although MFR testing operates at significantly lower shear rates, the presence of CNC particles may promote the formation of networks or agglomerates within the melt, which can increase the resistance to flow, effectively stiffen the composite material, and reduce the melt flow rate. All samples had a lower MFR than pure PHA. More specifically, PHA/0.5% CNC and PHA/1% CNC showed a gradual decrease in MFR. A moderate increase in the MFR of PHA/1.5% CNC was observed. Finally, the MFR decreased once again in samples with higher CNC loading, with the PHA/2.5% CNC presenting the lowest value.

The above findings are also in agreement with the results of the examination of the thermal properties of the various PHA/CNC nanobiocomposites. The IDT and FR, as determined by relevant TGA measurements, seemed to remain almost stable for the former and increased for the latter. The IDT at 95% seemed to be maximized for the PHA pure specimen, whereas the FR, as expected, steadily increased with increasing CNC content, and was maximized for the highest content of the PHA/CNC nanobiocomposite. Figure 4a,d presents the results of the TGA analysis, focusing on the Initial Decomposition Temperature (IDT) at 95%, which is the temperature at which the material loses 5% of its mass owing to thermal degradation, and the Fractional Residue (FR) percentage remaining after decomposition. The IDT_@95_ for all CNC-filled samples was lower than that of pure PHA and showed a slight decreasing trend as the CNC content increased. Because CNC thermally degrades at a lower temperature (200–240 °C) than PHA (∼270 °C), it serves as an early degradation site, reducing the overall IDT_@95_.^90^ However, at higher CNC loadings (2.5%), they may begin to form interconnected structures within the polymer matrix, which could delay early degradation. The FR increased with the CNC content, aligned with the increase in filler concentration.

The information derived from DSC measurements regarding the melting temperature and its evolution with increasing CNC content revealed that it remained practically unaffected by the addition of CNC into the PHA polymer matrix. This is also important for setting specific parameters during AM of various PHA/CNC nanobiocomposite specimens. Figure 4b,c shows the results of the DSC analysis. The heat required to melt the composites is shown in Figure 4b. Figure 4c presents the melting temperatures (*T*_m_) of all samples, which exhibit small fluctuations around the *T*_m_ of pure PHA, as fillers that do not directly affect *T*_m_ are introduced. These slight variations occur because CNC interfere with polymer chain packing at lower concentrations and act as nucleating agents.^91–93^ According to Mariano and Dufrense,^94^ the *T*_m_ of CNC exceeds the degradation temperature. The glass transition temperature (*T*_g_) was not visible as the PHA concentration was lower than the range of the temperature conditions of the experiment.^95^

The storage and loss modulus behavior with increasing temperature showed a decrease for the former and an increase for the latter, as expected, whereas the glass transition temperature, as extracted by the tan­(δ) behavior, seemed to be maximum for the PHA pure polymer, whereas it fluctuated with a diminishing trend for all other PHA/CNC nanobiocomposites. This could be the result of the addition of more crystalline phases into the PHA polymer matrix, as *T*_g_ decreased with increasing crystalline phases of the polymers.^96^

Figure 5 illustrates the storage modulus, loss modulus, and (tan­(δ)) as functions of the temperature. The storage and loss moduli of the composites decreased compared to those of pure PHA, indicating weakening of the samples. Finally, the peaks of tan­(δ) were close to the melting temperatures observed in the DSC analysis because of the high crystallinity of the PHA polymer and decreased with increasing filler concentration. Figure 5f shows that the aforementioned moduli and tan­(δ) digress from the trend and increase significantly compared with those of the other composites. Again, this can be attributed to potential agglomeration in the case of high CNC loading, leading to polymer chain mobility restriction.

The most interesting picture of the various mechanical properties of the prepared PHA/CNC nanobiocomposites was the flexural and tensile strength and elasticity of the parts, which seemed to become optimum around the 0.5 wt % CNC filler content in the PHA polymer matrix. Above this compositional mark, both properties seemed to diminish, probably owing to the particle clustering phenomena. On the other hand, the flexural and tensile toughness seemed to diminish with increasing CNC filler content for low CNC concentrations but seemed to quickly pick up maximizing for the highest CNC filler content PHA/CNC nanobiocomposite. This is probably due to agglomeration between the CNC, which creates larger “superstructures” that favor flexural and tensile toughness. From the above experimental results, the most favorable property seems to be the increase in the tensile modulus of elasticity, which seems to increase by an impressive 47.3% with respect to pure PHA in the 0.5 wt % compound.

In contrast, the toughness of the filaments was maximized for the PHA pure specimen, whereas it diminished significantly for all the other PHA/CNC nanobiocomposites. This could be because of the treatment after heating and melting from the nozzle during the 3D printing of the samples, which possibly relieves internal stresses and causes the final material to exhibit a different behavior after being 3D-printed. The same was observed for the Charpy Impact strength, which is maximum for the PHA pure specimen and diminishes rapidly upon the addition of CNC into the PHA pure polymer matrix. Analogous behavior, as expected, was exhibited by Vickers microhardness, which was also found to maximize PHA purity, while it diminished steadily with increasing CNC filler content, minimizing the highest CNC content in the PHA/CNC nanobiocomposite. The 0.5 wt % compound demonstrated superior mechanical strength and stiffness compared to the other loadings tested. However, these nanocomposites showed low toughness values, indicating that the increase in the strength and stiffness of the PHA biopolymer due to the introduction of the CNC nanoparticles led to a more brittle response and decreased the ability of the PHA biopolymer to absorb energy when loads were applied to it. The increase in tensile strength was not impressive; however, the material became considerably stiffer.

The integration of cellulose nanocrystals (CNCs) into polyhydroxyalkanoates (PHAs) matrices for material extrusion (MEX) 3D printing typically leads to a notable enhancement in Young’s modulus, a slight increase in tensile strength, and a simultaneous decrease in both impact strength and microhardness, as found in the experiments conducted herein. These nanocomposites characteristics are attributed to the intrinsic properties of CNCs and the polymer matrix. CNCs exhibit a high aspect ratio, high crystallinity, and significant stiffness (modulus ∼70 GPa^97^), which contribute to the increased stiffness of the nanocomposite, through a mechanism known as stress transfer between the matrix and the nanofillers.^98^ The modest increase in tensile strength suggests inadequate interfacial adhesion between the polymer and nanofillers or, potentially, the aggregation of CNCs within the PHA matrix (which could not be verified herein through SEM, due to the nature of the filler), where such agglomeration could act as a stress concentration point, impeding the effective load transfer necessary for enhanced strength.^99^ Beyond 0.5 wt.% concentration, the mechanical properties start to decline, which indicate saturation of the filler in the matrix. Furthermore, the reduction in impact strength is attributed to CNCs restricting polymer chain mobility, thereby reducing the matrix’s ductility. This was verified in the experiments, in which in introduction of CNCs in the PHA led to samples’ failure at lower strain. Additionally, possible CNC agglomerates can form rigid or brittle zones.^100^ These stiff interfaces limit the plastic deformation involved in fracture propagation, increasing the likelihood of crack initiation under loads. Therefore, increased brittleness leads to a decline in the impact strength. Moreover, the decreased microhardness indicates localized stress concentrations at the filler–matrix interface and potential microstructural discontinuities at filler–polymer matrix interfaces, where poorly dispersed CNCs and weak interfacial bonding significantly impair resistance to localized indentation forces.^101,102^ Thus, although CNCs enhance the elastic modulus due to their rigid properties, they negatively impact toughness-related properties unless improved surface modification or dispersion techniques are employed to enhance their compatibility with the hydrophobic PHA matrix. Such techniques were not applied as they were not within the scope of the research, which focus on the effect of CNCs on the properties of PHA within the context of MEX 3D printing. This aimed at introducing new biodegradable and eco-friendly nanocomposites in MEX 3D printing, in an effort to increase its sustainability, through the reduction of petroleum-based polymers. To make that possible, nature sourced materials should provide adequate mechanical performance but also, thermal stability, ease of processability, and overall good properties and behavior when producing parts with the MEX 3D printing method. The improved stiffness of the CNCs induced to the PHA is a sought-after characteristic in various applications. In combination with the improved strength (especially in flexural loads) make these nanocomposites valuable for real-life applications. On the other hand, the reduced impact strength and the more brittle behavior are actual drawbacks of the nanocomposites, limiting their compatibility with applications involving dynamic or impact use. This should be considered when designing parts to be built with such nanocomposites. The reduced microhardness also suggests that these nanocomposites are not suitable in applications requiring high wear resistance from the materials.

The HD μ-CT findings showed that for moderate concentrations of CNC, namely the PHA/1 wt % CNC nanobiocomposite, both the porosity and the dimensional deviation seemed to become minimum, by 20.6% for the former and 18.4% for the latter, with respect to pure PHA. It is very important to consider 3D printed samples, as these nanobiocomposites have very important applications as biomaterials. As previously stated, dimensional accuracy plays a crucial role. The same is true for porosity, and a high porosity is directly linked to the possible mechanical failure of the material, which is a very problematic situation, especially for its possible use as a biomaterial.

The broad investigation protocol was completed through SEM investigation of both side faces of the 3D printed examples and the fractured cross sections of the specimens during mechanical testing. These images clearly show that upon the addition of CNC, the polymer becomes very smooth on its surface, indicating that the incorporation of CNCs into the PHA pure polymer matrix enhances the overall quality of the final PHA/CNC nanobiocomposites. Again, the most favorable CNC concentration appeared to be moderate, as shown in the SEM images in Figure 10. The lateral SEM images show a uniform layer thickness and good fusion between the layers without defects in all the prepared compounds. The lack of significant differences in the structure was expected because the rheological properties did not significantly differ between the compounds with different CNC loadings. The inspection of the fracture region showed a rather brittle fracture mechanism in all samples, with low deformation on the 3D printing structure during its failure in the tensile experiment.

Overall, the incorporation of CNC into the PHA pure polymer matrix seems to be beneficial for the mechanical, rheological, thermal, and structural properties of the corresponding PHA/CNC nanobiocomposites, which could open a way for more detailed applications of the relevant materials toward a more sustainable future for the use of polymers with low environmental impact. In the literature, no nanocomposites similar to nature-sourced materials have been found to compare current research outcomes. As the composite preparation process affects their performance, compared with composites developed using other methods and techniques, it is not reliable. The effect of CNC on (PCL) in the bioplot^103^ was similar to the current findings. The tensile strength increased more in this case than that in the current study, whereas the flexural strength increased more in the current study. The composites were prepared using a thermomechanical extrusion process similar to that used in this study. Cellulose nanofibers improve the mechanical strength of polycarbonate by approximately 10% in MEX AM.^31^ In this study, the composites were prepared using a thermomechanical extrusion process similar to that used in the current study. The tensile and flexural strengths achieved in the current study are still lower than those of popular petroleum-based polymers such as PLA^104^ in MEX AM. Nevertheless, the nature-sourced, biocompatible, and eco-friendly characteristics of the proposed nanocomposites justify (at least partly) their merits as alternatives to common petroleum-based polymers.

## 5 Conclusions

In this study, we thoroughly examined the effect of incorporation of CNC into a PHA polymer matrix on the mechanical, rheological, thermal, and structural properties of the corresponding PHA/CNC nanobiocomposites within the MEX 3D printing context. The aim was to produce materials for the MEX AM method with minimum environmental impact and good performance. A series of specimens with different compositions and CNC loadings were prepared. The loadings were 0.5, 1.0, 1.5, 2.0, and 2.5 wt % CNC. The main findings can be summarized as follows:

- The improvement of the various properties cumulatively occurs for moderate CNC filler content in the PHA pure polymer matrix, namely 0.5 wt % CNC, which was found to be overall the optimum loading.
- The tensile and flexural strength improved 8.6 and 23.3% respectively, compared to the unfilled PHA.
- The improvement in stiffness was significant, with 47.3 and 20.3% increase in the Young’s and flexural modulus respectively, compared to the unfilled PHA.
- On the other hand, the impact strength and microhardness decreased, which should be considered when designing parts to be built with these specific nanocompounds.
- The quality metrics (porosity and geometrical accuracy) improved by the addition of the CNC in the PHA matrix.
- The thermal and rheological metrics were not highly affected.

Overall, the nature-sourced biocompatible and eco-friendly PHA/CNC composites developed for the MEX AM process were proven to have sufficient strength and characteristics for use as alternative materials to conventional petroleum-based polymers for the fabrication of parts using this method.

Further investigations would involve the incorporation of another filler content that has been shown to improve the impact strength in other cases, such as silica fillers,^105^ or by inducing antibacterial properties.^106^ This would open new pathways in multifunctional material engineering, with even more practical applications in areas where polymers do not usually find any major applications, such as defense or space.

## Supplementary Material



## Data Availability

The raw/processed data required to reproduce these findings cannot be shared because of technical or time limitations.

## Notes

Filament evaluation using a microscope (Kern OKO 1), quality control, and mechanical properties (Figure S1); stress vs strain curves (Figure S2); 3D printed samples and settings for their fabrication (Figure S3); microcomputed tomography results for pure PHA and the prepared nanocomposites (0.5–2.5 wt %) (Figure S4); spider-type graphs summarizing the main experimental findings for pure PHA and the nanocomposites (0.5–2.5 wt %) (Figure S5) (PDF)
