# Supplementary material for: Reinforced PHA/CNC Biocomposites in Extrusion-Based Additive Manufacturing
Source: ACS Omega. 2025 Aug 5;10(32):36613–30. doi: 10.1021/acsomega.5c05743 (PMC12368640; doi:10.1021/acsomega.5c05743)
Supplement: Supplementary file 1 [file ao5c05743_si_001.pdf]

Reinforced PHA/CNC biocomposites in extrusion-based additive manufacturing

Markos Petousis [a], Constantine David [b], Dimitrios Sagris [b], Nektarios K. Nasikas [c], Vassilis Papadakis [d, e], Apostolos Argyros [f, g], Vasileios Stratiotou Efstratiadis [f, g], Aikaterini Gaganatsiou [a], Nikolaos Michailidis [f, g], and Nectarios Vidakis [a*]

[a] Department of Mechanical Engineering, Hellenic Mediterranean University, Heraklion 71410, Greece, markospetousis@hmu.gr; gkagka@hmu.gr; vidakis@hmu.gr;

[b] Dept. of Mechanical Engineering, International Hellenic University, Serres Campus, 62124, Greece, david@ihu.gr; dsagris@ihu.gr;

[c] Division of Mathematics and Engineering Sciences, Department of Military Sciences, Hellenic Army Academy, 16673 Vari, Attica, Greece, nasikas@sse.gr

[d] Institute of Electronic Structure and Laser of the Foundation for Research and Technology-Hellas (IESL-FORTH) – Hellas, N. Plastira 100m 70013 Heraklion, Greece

[e] Department of Industrial Design and Production Engineering, University of West Attica, 122 43 Athens, Greece, v.papadakis@uniwa.gr

[f] Physical Metallurgy Laboratory, Mechanical Engineering Department, School of Engineering, Aristotle University of Thessaloniki, 54124 Thessaloniki, Greece, aargyros@auth.gr; vstratio@meng.auth.gr; nmichail@auth.gr;

[g] Centre for Research & Development of Advanced Materials (CERDAM), Centre for Interdisciplinary Research and Innovation, Balkan Centre, Building B', 10th km Thessaloniki-Thermi road, 57001, Thessaloniki, Greece

[*] Corresponding author: e-mail: vidakis@hmu.gr (Nectarios Vidakis), Tel.: +302810379227

Abstract

Polyhydroxyalkanoate (PHA) is a biopolymer that can be 3D printed using the material extrusion method. Nevertheless, their mechanical properties are inferior to those of petroleum-derived polymers, which restricts their broader application. Herein, nanobiocomposites comprising naturally sourced PHA and cellulose nanocrystals (CNC) as fillers were successfully synthesized. These nanobiocomposites were prepared with filler concentrations ranging from 0.5% to 2.5% by weight, in increments of 0.5 wt. %. Filaments were produced from binary PHA/CNC mixtures and subsequently employed for 3D printing of the respective nanobiocomposite samples. They were subjected to mechanical, rheological, thermal, and structural analyses using analytical techniques and the respective standards. The integration of CNC into the pure PHA polymer matrix has been reported to enhance PHA's mechanical properties of PHA, with an increase in flexural strength by 23.3%, flexural modulus by 20.8%, and Young's modulus by 47.3%, although there was a reduction in impact strength and microhardness. Morphological characterization confirmed the homogeneous dispersion of CNC, whereas the thermal and rheological properties remained almost unchanged. The porosity

and geometric accuracy of the 3D-printed samples, evaluated using micro-CT, were improved by incorporating CNC into the PHA matrix. The 0.5 wt% CNC concentration was the optimum one, improving mechanical and quality metrics. These findings highlight the potential of PHA/CNC nanocomposites as innovative high-performance biodegradable materials for 3D printing for biomedical, packaging, and structural engineering applications. Such nanobiocomposites can contribute to reducing the environmental impact of petroleum polymers through a cost-effective additive manufacturing method.

S.1. Raman spectroscopy, rheology, and thermal properties

The Raman spectra were acquired using a LabRAM HR Raman Spectrometer (HORIBA, Kyoto, Japan) equipped with a 532 nm solid-state laser, capable of a maximum output power of 90 mW. The spectral resolution was approximately 2 cm^{-1}, facilitated by a grating with 600 grooves. An Olympus objective lens (LMPlanFL N) with a numerical aperture of 0.5 was employed to deliver light and collect Raman signals. The lens, with a 50X magnification, had a working distance of 10.6 mm. A Neutral Density filter with 3.2% transmittance was used to limit the laser power to 1.2 mW for the sample. The measurement volume was defined as 1.7 μm laterally and 2 μm axially. Raman spectra were recorded in the range of 50 to 3900 cm^{-1}, utilizing three optical windows. Each data point was exposed for 10 seconds, with five accumulations. Visual inspection of the areas ensured no discoloration or degradation occurred due to laser irradiation.

The raw Raman data were processed using LabSpec software (HORIBA, Kyoto, Japan). Each spectrum underwent the following processing steps: a) removal of cosmic rays; b) denoising with a 5-point kernel; c) background removal using an 8th-grade polynomial; d) recalibration based on the 810 cm^{-1} peak; e) normalization by the maximum peak.

Rheological assessments were carried out on all filaments (pure PHA and composites), using Discovery Hybrid Rheometer DHR20 from TA Instruments (New Castle, DE, USA). A parallel plate configuration was employed, accompanied by a heating system capable of functioning across a temperature spectrum of -160°C to 600°C. The experiments were specifically performed at 190°C. The rheometer operated within a torque range spanning 1 to 100,000 μN·m, utilizing 25 mm plates with a 1 mm gap [1]. Melt Flow Rate (MFR) was assessed to characterize the flow properties of the composite materials under standardized conditions (ASTM D1238-13) at 190°C, with results expressed in grams per 10 minutes.

Thermogravimetric analysis (TGA) was conducted with the Discovery Simultaneous Thermal Analyzer SDT 650 from TA Instruments (Delaware, USA). This system is capable of operating from ambient temperature up to 1500°C, with a heating rate adjustable between 0.1 and 100°C/min and a precision of ±0.5% [2]. The testing protocol began at 25°C, gradually increasing to 450°C.

Differential Scanning Calorimetry (DSC) was performed with Discovery-Series DSC 25 from TA Instruments (Delaware, USA), which features an integrated cooling system and supports temperature measurements within a range of −90°C to 725°C, with heating rates from 0.01 to 100°C/min [3]. DSC analysis was designed to assess the thermal properties of the composite filaments, following a controlled heating sequence starting at 25°C and reaching 250°C.

S.2. Filament testing

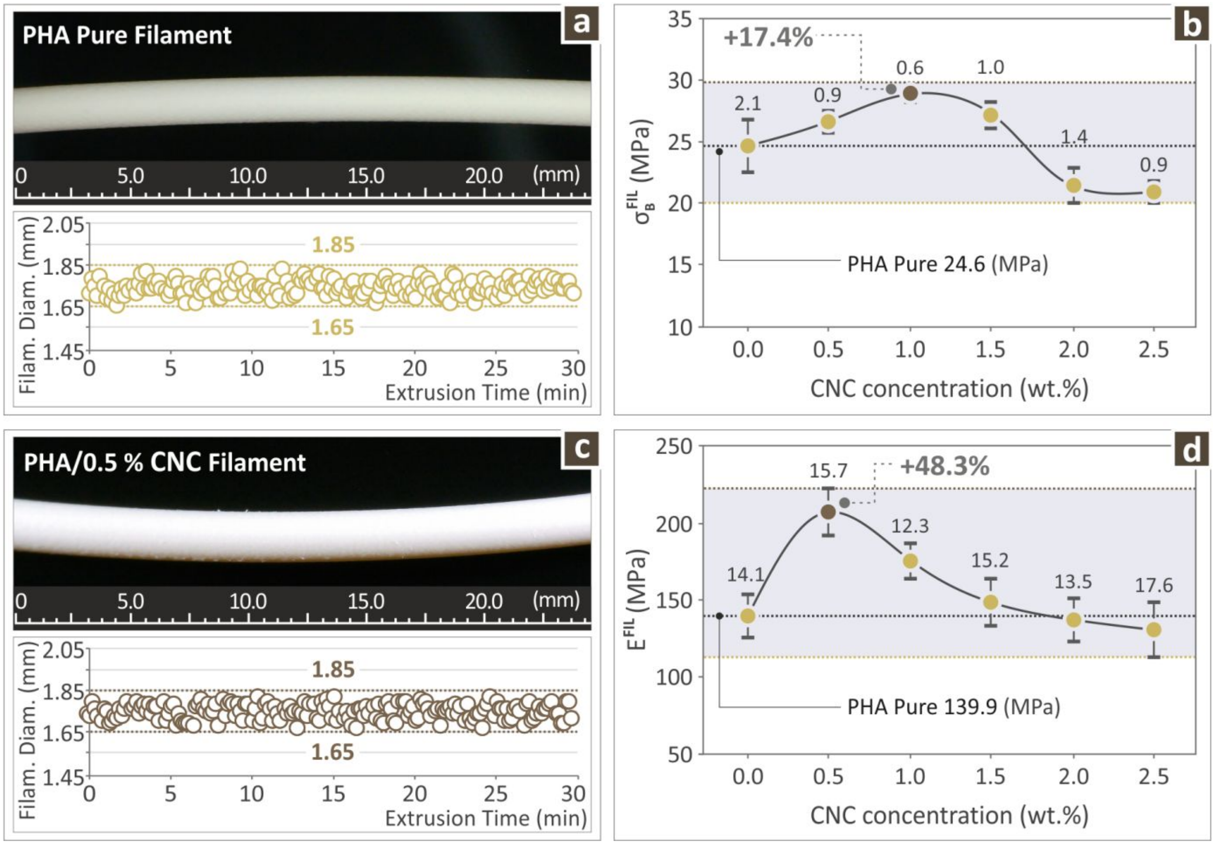

Figure S1. Filament evaluation using a microscope (Kern OKO 1), quality control, and mechanical properties: (a) inspection of PHA pure filament under the microscope and diameter variation during its production, (b) tensile strength of the filaments tested (pure and nanocomposites), (c) inspection of PHA/CNC 0.5 wt. % nanocomposite filament under the microscope and diameter variation during its production, (d) Young's modulus of the filaments tested (pure and nanocomposites)

S.3. Stress vs. strain graphs

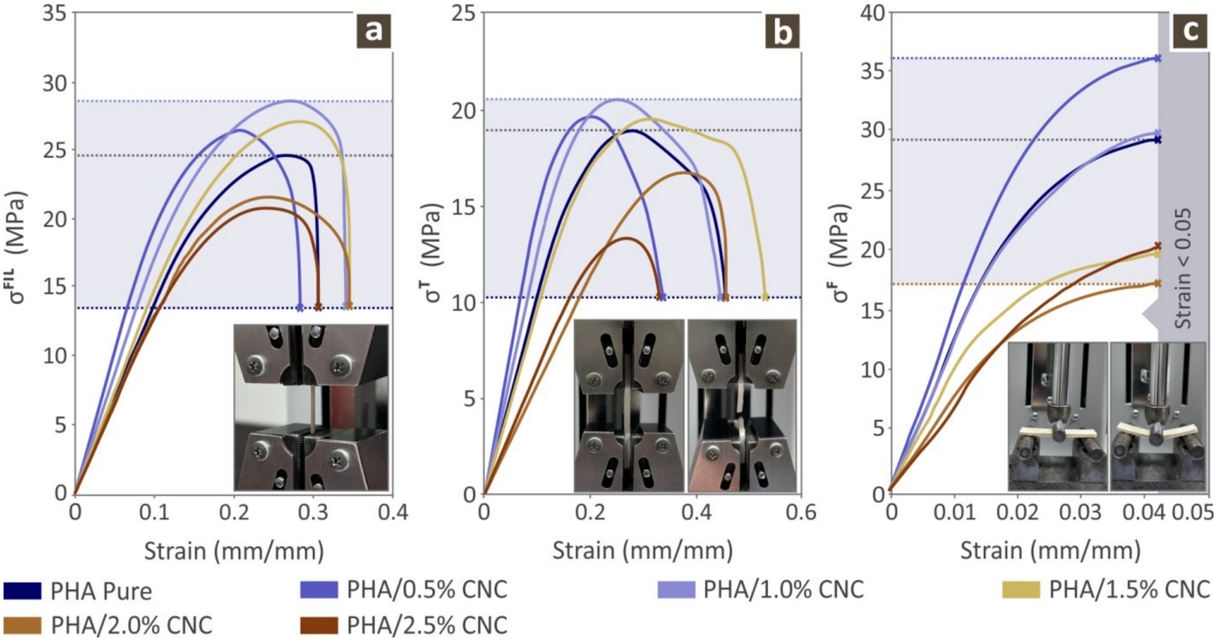

Figure S2. Stress vs. strain curves for (a) filament, and 3D printed samples (b) tensile test and (c) flexural (three-point-bending) test

S.4. 3D printed samples and settings for their fabrication

Parameters	Value	Units
PHA CNC content	0.0–0.5–1.0–1.5–2.0–2.5	%
Printing orientation	±45	deg
Nozzle temperature	200	°C
Bed temperature	40	°C
Layer thickness	0.2	mm
Number of perimeters	2	–
Fill density	100	%
Travel speed	40	mm/sec

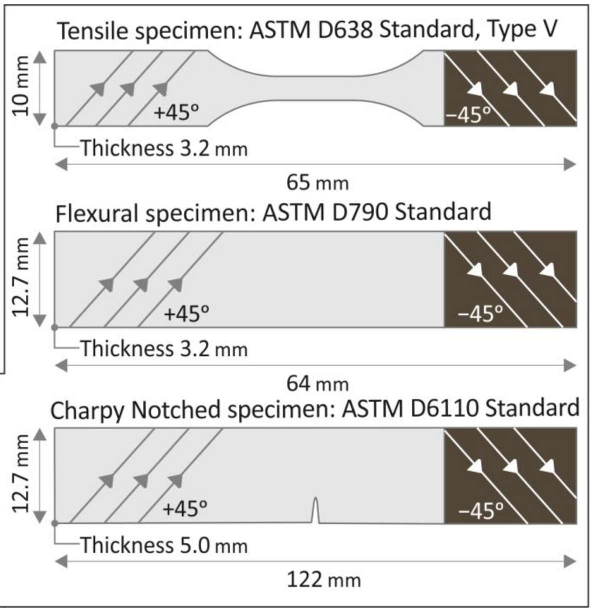

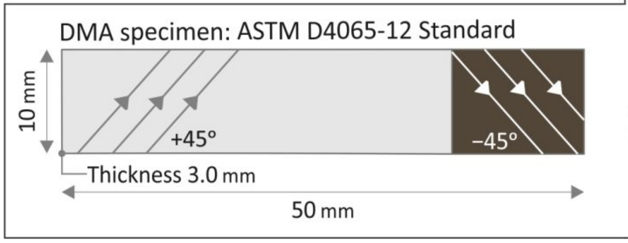

Figure S3. 3D printed settings were utilized for pure PHA and PHA/CNC nanocomposites (same settings for all, for comparison) and the geometry of the fabricated mechanical test samples according to the respective standards for each test. The raster orientation of the 3D structure is also depicted.

S.5. Dimensional deviation and porosity findings

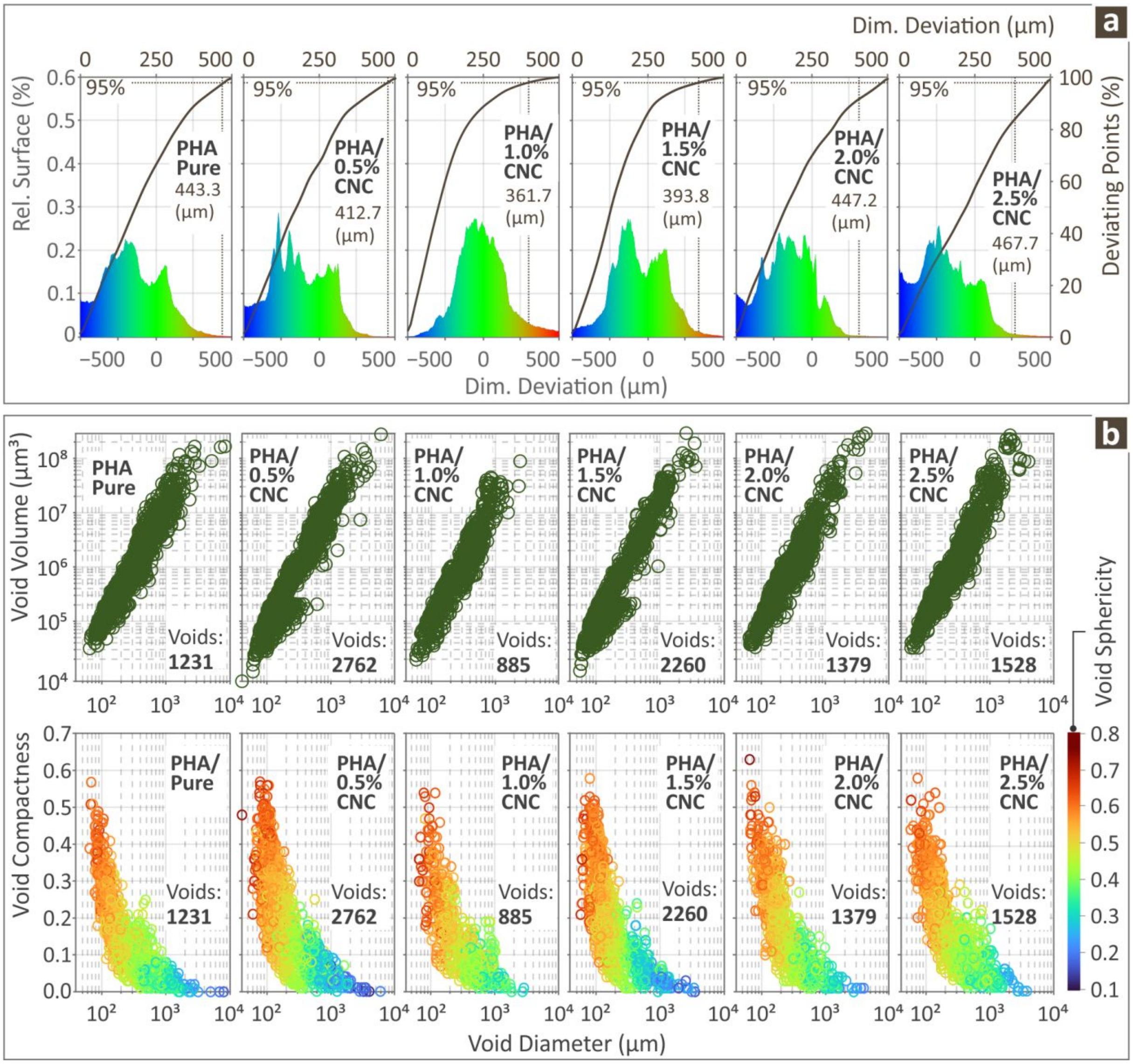

Figure S4. Micro-computed tomography results for pure PHA and the prepared nanocomposites (0.5-2.5 wt. %) (a) dimensional accuracy, (b) voids volume, compactness, and sphericity

S.6. Summary of the main experimental findings

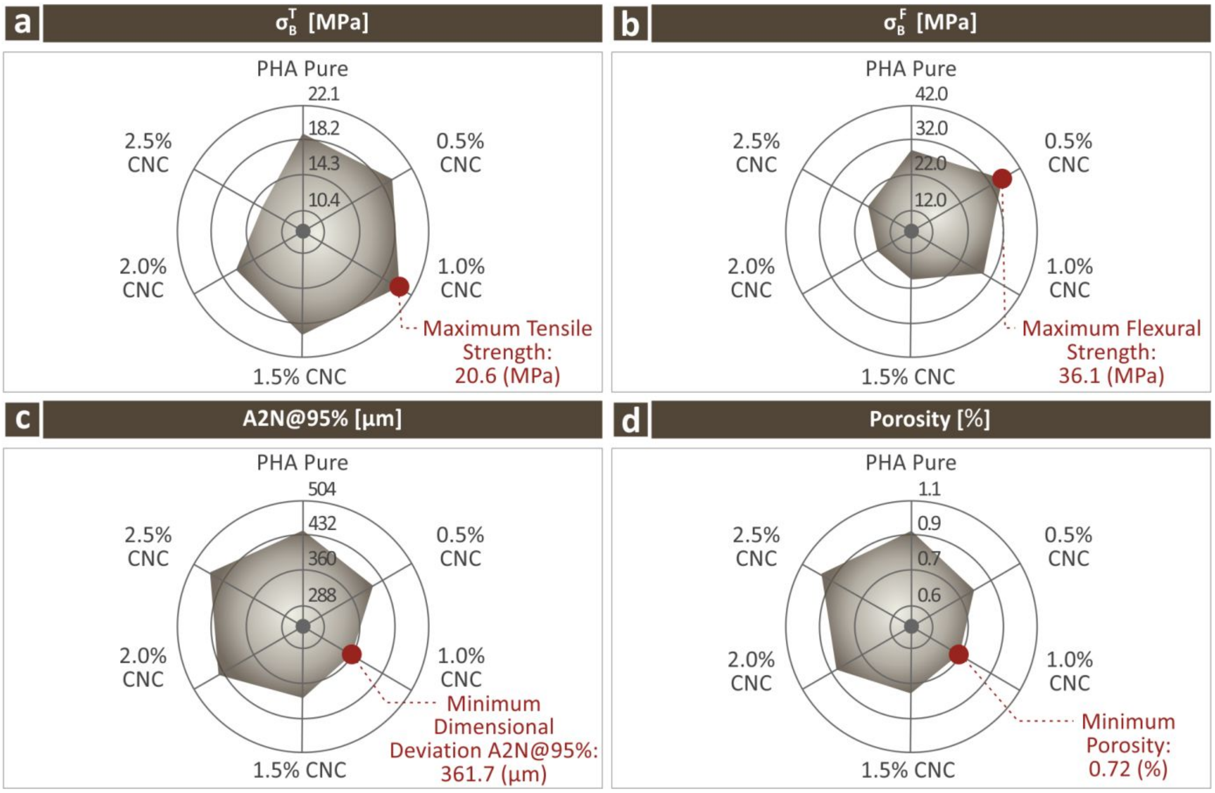

Figure S5. Spider-type graphs summarizing the main experimental findings for pure PHA and the nanocomposites (0.5-2.5 wt. %) (a) tensile strength, (b) flexural strength, (c) dimensional deviation, (d) porosity.

References

(1) Corporation, T. I. *TA Instruments (Rheometer)*. https://www.tainstruments.com/hr-20/ (accessed 2025-03-12).

(2) *SDT 650 TA Instruments*. https://www.tainstruments.com/sdt-650/ (accessed 2025-03-12).

(3) *TA instruments (DSC)*. https://www.tainstruments.com/hr-20/ (accessed 2025-03-12).
